# Stability and inter-family associations of hair endocannabinoid and *N*-acylethanolamines across the perinatal period in mothers, fathers, and children

**DOI:** 10.1038/s41598-024-59818-6

**Published:** 2024-04-24

**Authors:** L. Bergunde, S. Steudte-Schmiedgen, M. Karl, I. Jaramillo, W. Gao, T. von Soest, S. Garthus-Niegel

**Affiliations:** 1 Institute and Policlinic of Occupational and Social Medicine, Faculty of Medicine, Technische Universität Dresden, Dresden, Germany; 2 Department of Psychotherapy and Psychosomatic Medicine, Faculty of Medicine, Technische Universität Dresden, Dresden, Germany; 3 School of Psychology, Nanjing Normal University, Nanjing, China; 4 Department of Psychology, PROMENTA Research Center, University of Oslo, Oslo, Norway; 5 Institute for Systems Medicine (ISM), Faculty of Medicine, Medical School Hamburg MSH, Hamburg, Germany; 6 Department of Childhood and Families, Norwegian Institute of Public Health, Oslo, Norway

**Keywords:** Developmental biology, Psychology, Biomarkers

## Abstract

Analysis of endocannabinoids (ECs) and *N*-acylethanolamines (NAEs) in hair is assumed to retrospectively assess long-term EC/NAE concentrations. To inform their use, this study investigated stability of EC/NAE hair concentrations in mothers, fathers, and their children across the perinatal period as well as associations between family members. In a prospective cohort study, EC (AEA, 1-AG/2-AG) and NAE (SEA, PEA, OEA) levels were quantified in hair samples taken four times in mothers (*n* = 336) and their partners (*n* = 225) from pregnancy to two years postpartum and in offspring (*n* = 319) from shortly after birth to two years postpartum. Across the perinatal period, maternal and paternal hair ECs/NAEs showed poor multiple-test consistency (16–36%) and variable relative stability, as well as inconsistent absolute stability for mothers. Regarding children, hair ECs/NAEs evidenced poor multiple-test consistency (4–19%), no absolute stability, and either no or variable relative stability. Hair ECs/NAEs showed small to medium significant associations across the perinatal period within couples and parent–child dyads. Findings suggest hair ECs/NAEs during the perinatal period possess variable stability in adults, albeit more stability in fathers than mothers in this time. This highlights the need to further investigate factors associated with changes in hair ECs/NAEs across time. The first two years of life may be a dynamic phase for the endocannabinoid system in children, potentially characterized by complex within-family correspondence that requires further systematic investigation.

## Introduction

The analysis and quantification of hormones and ligands in hair has emerged as an important methodological addition to the psychoneuroendocrine research toolbox^1,2^. In particular, hair cortisol concentrations (HCC) represent the most established hair-based marker to date, showing utility for clinical diagnostics, therapy monitoring, and mental health and stress research^1^. It is assumed that hormones and ligands are incorporated in growing hair and consequently hair concentrations retrospectively reflect cumulated secretion of the respective hormone or ligand over several months^3^. An implicit assumption underlying hair analysis is that in the absence of major life events, hair concentrations remain largely stable within an individual^2^. HCC have been intensively investigated and validated, with studies documenting a considerable degree of intraindividual stability of HCC in community samples^4–6^ and postpartum in mothers and infants^7^, but not in expectant mothers across pregnancy^8^. Taken together, findings underscore HCC as a useful biomarker estimate of long-term cortisol secretion and a key variable in chronic stress studies. However, evidence for other hair-based biomarkers remains limited in this respect.

This is particularly relevant for the assessment of the ligands of the human endocannabinoid system (ECS), the endocannabinoids (ECs), to better understand the role of EC signalling in health and disease^9^. Acute circulating EC levels have frequently been measured in blood plasma and serum^10–12^, and have recently also been quantified in human hair using liquid chromatography-tandem mass spectrometry^13,14^. The ECS is a neuromodulatory system which regulates multiple physiological functions in the body ranging from the central nervous system to the reproductive system^15,16^. Amongst these, the ECS has been shown to interact with the hypothalamus–pituitary–adrenal (HPA) axis, assuming an important regulatory function under basal and stressful conditions^17^. ECs are lipid signalling molecules synthesized mainly on demand that act primarily as retrograde messengers via presynaptic cannabinoid receptors (CB1 and CB2)^18^. The main ECs and best studied in terms of their biologic functions are anandamide (AEA^19^) and 2-arachidonoylglycerol (2-AG^20^), while additional *N*-acylethanolamines (NAEs) exist, among others stearoylethanolamide (SEA), palmitoylethanolamide (PEA), and oleoylethanolamide (OEA), that exert their respective biological activities through different receptors^21,22^.

Quantifying ECs/NAEs in human hair has the potential to provide a retrospective picture of long-term peripheral EC/NAE levels over several months and additionally involves a non-invasive and stress-free sampling process^14,23^. Similar to HCC, the most common hypothesis for incorporation of ECs/NAEs in hair is based on the multi-compartment model^24^. Incorporation is suggested to occur mainly 1) during hair shaft formation via diffusion from blood circulation, 2) after formation via sweat and sebum glands or from surrounding tissues, and also 3) after hair has emerged from the skin via the external environment^25^. Basic lipophilic compounds, such as AEA and NAEs, are assumed to mainly be incorporated into hair via the bloodstream, whereas 2-AG, with its neutral chemical structure, is likely incorporated via sweat and sebum^26^.

Several studies have employed hair analyses of ECs/NAEs to examine their role in the pathophysiology of (stress-related) mental disorders, such as depression^27^, (childbirth-related) posttraumatic stress disorder^28,29^, cocaine use disorder^30^, anorexia nervosa^31^, anxiety, and burnout^14^. In these studies, hair ECs/NAEs were employed to assess ECS activity over an extended time up to 3 months, implicitly assuming that in the absence of major life events hair EC/NAE concentrations are somewhat stable within an individual, i.e., reflect a stable factor. However, this assumption has been insufficiently validated to date. Only one study systematically examined this for hair ECs/NAEs, finding high intra-individual stability of hair ECs/NAEs in a community sample of 100 female adults assessed across 2.5 years^32^. While these initial results suggest ECs/NAEs measured in hair may represent biomarkers that remain largely stable across time, findings are not generalizable to males or children and the study did not actively control for major life events during the 2.5-year study period.

Hence it remains to be tested whether hair ECs/NAEs show intraindividual stability and represent stable biomarkers also in the presence of a major life event. One possibility to examine this is to look at how the experience of a particular major life event, such as pregnancy and childbirth, affects intraindividual stability of hair ECs/NAEs in mothers and fathers. From a developmental perspective, pregnancy and the birth of a child represent a major life event as they represent a time-discrete transition in the parents’ lives that impacts subjective well-being and requires adaptation processes in the short- and long-term^33^. Furthermore, pregnancy and childbirth involve complex physiological mechanisms that include many processes that are part of the stress response, with childbirth involving extreme physical stress^34^. For (expectant) mothers, pregnancy and childbirth go hand in hand with major physiological changes^35^, including changes to endocannabinoid and cortisol signalling^36^ and HPA axis functioning^37^, with HCC having been found to increase across pregnancy and subsequently decrease postpartum^38^. Some physiological changes have also been observed in (expectant) fathers (e.g., decline in testosterone^39–41^). Thus, available research supports pregnancy and childbirth as a major life event^33^ that may involve psychological and physiological stress for (expectant) parents^42^ and consequently it remains to be tested how stable hair EC/NAE measures are in this time in mothers and fathers.

Furthermore, while evidence regarding a role for the ECS in child and neural development is accumulating^43,44^, to our knowledge only one study has investigated hair ECs/NAEs in children aged up to one year^45^. They found no association between child hair ECs/NAEs in utero (i.e., assessed via hair taken shortly after birth) and at one year after birth, indicating low stability and further reported an increase in 1-AG/2-AG and a decrease in NAEs in this time. Regarding HCC as another hair-based marker in children, the effect of age has been mixed^46^, but studies did find that HCC correlated positively across time up to the age of 8 years^47,48^. These HCC findings indicate a stable component, consistent with findings of intraindividual stability of salivary cortisol in the first year of life^49^. Hence, investigating whether and how hair EC/NAEs change from birth onwards is warranted to inform our understanding of the ECS in child development as well as the utility of hair EC/NAE levels as a potential biological indicator to study child health.

This study therefore primarily aimed to investigate multiple-test consistency, relative stability, and absolute stability for maternal, paternal, and child hair ECs/NAEs across the perinatal period. Regarding parents, we examined these measures of stability when a major life event (i.e., pregnancy and childbirth) was included and when it was not (i.e., examining only the later postpartum period from 14 to 24 months postpartum) and expected for mothers that multiple test consistency and relative and absolute stability in hair ECs/NAEs would differ between these two conditions, as previous evidence suggests alterations in hair ECs/NAEs during pregnancy in (expectant) mothers^36^. Due to scarce research on paternal hair ECs/NAEs during the perinatal period, no specific predictions were formulated for fathers. We further expected parental hair ECs/NAEs to show at least moderate multiple-test consistency and test–retest stability, but as findings regarding absolute stability have been inconsistent^32^, no specific hypotheses were formulated. Furthermore, due to insufficient evidence regarding children’s hair ECs/NAEs, no specific hypotheses regarding their stability were posed.

Finally, only two studies have to date examined the relation between hair ECs/NAEs in children and maternal hair ECs/NAEs sampled shortly after birth^50^ and at one year postpartum^45^, finding no consistent relationships between maternal and child hair ECs/NAEs. Findings regarding a more established biomarker in hair (i.e., HCC) indicate a positive relationship that attenuates with increasing age^51–53^, but see^7,54^ for no significant relationship. Consequently, we aimed to clarify these heterogeneous previous findings by investigating how maternal and child hair EC/NAE concentrations relate to one another up to two years after birth. Furthermore, to expand on prior research we also examined relationships between children’s and fathers’ and fathers’ and mothers’ hair EC/NAE concentrations as a positive relationship has been reported regarding HCC^55^.

## Methods

### Study design and participants

**Figure 1:**
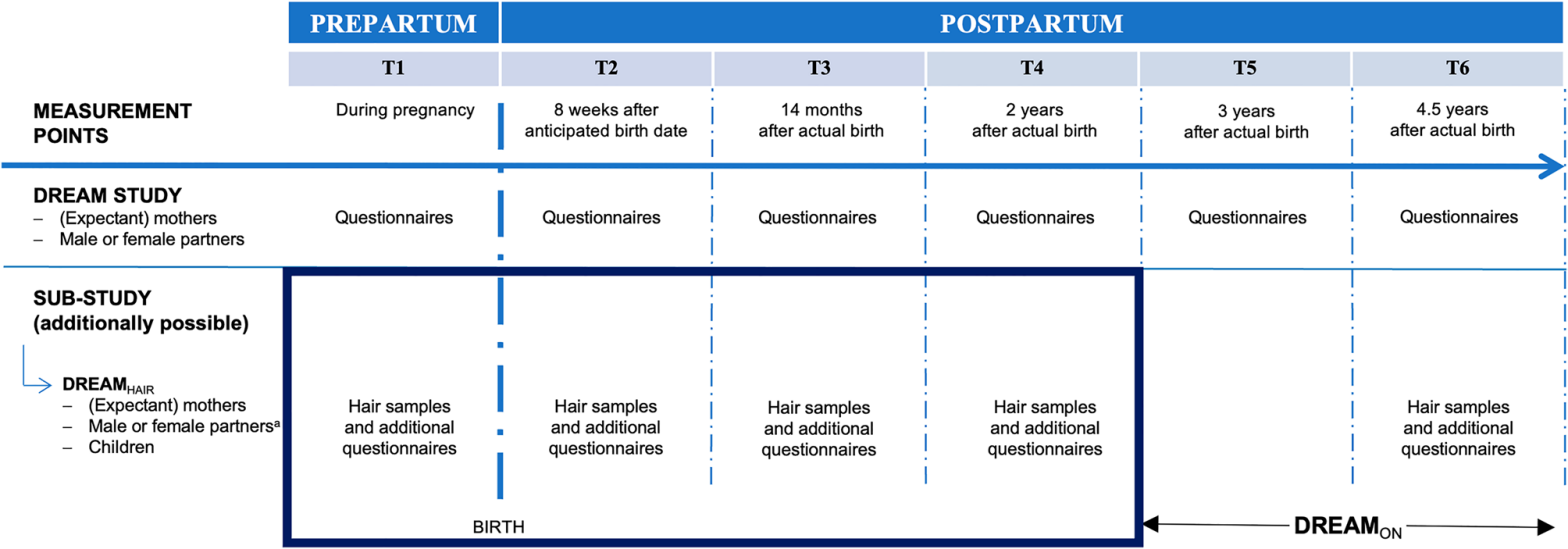
Assessment waves of the DREAM Study (prolongation into middle childhood planned) and its endocrine sub-study DREAM_HAIR_. *Note.* Boxed assessments are relevant to this investigation. This study used hair samples and DREAM_HAIR_ questionnaires for the measurement points T1 (during pregnancy or shortly after birth; mothers: *M* = 21.47 days before birth, *SD* = 10.80; fathers: *M* = 18.64 days before birth, *SD* = 11.15; children: *M* = 9.82 days after birth, *SD* = 5.63), T2 (8 weeks after the anticipated birth date; mothers: *M* = 8.43 weeks after birth, *SD* = 1.38; fathers: *M* = 8.37 weeks after birth, *SD* = 1.34; children: *M* = 8.48 weeks after birth, *SD* = 0.81), T3 (14 months postpartum; mothers:* M* = 13.89 months after birth, *SD* = 0.64; fathers: *M* = 13.88 months after birth, *SD* = 0.64; children:* M* = 13.88 months after birth, *SD* = 0.57), and T4 (24 months postpartum; mothers: *M* = 23.94 months after birth, *SD* = 0.64; fathers: *M* = 23.96 months after birth, *SD* = 0.62; children: *M* = 23.90 months after birth, *SD* = 0.62) ^a^for the present investigation female partners (n = 2) were pre-emptively excluded.

Participants of this study took part in the Dresden Study on Parenting, Work, and Mental Health (DREAM) and its endocrine sub-study DREAM_HAIR_^56^, a large prospective cohort study approved by the ethics committee of the *Technische Universität Dresden* (EK 278062015). The study was conducted in accordance with the Declaration of Helsinki. DREAM_HAIR_ is a multi-method study investigating long-term biological determinants of perinatal stress and mental health-related outcomes in mothers, their partners, and their offspring across five measurement points from birth to 4.5 years postpartum (T1 DREAM_HAIR_–T6 DREAM_HAIR_, see Fig. 1).

**Figure 2:**
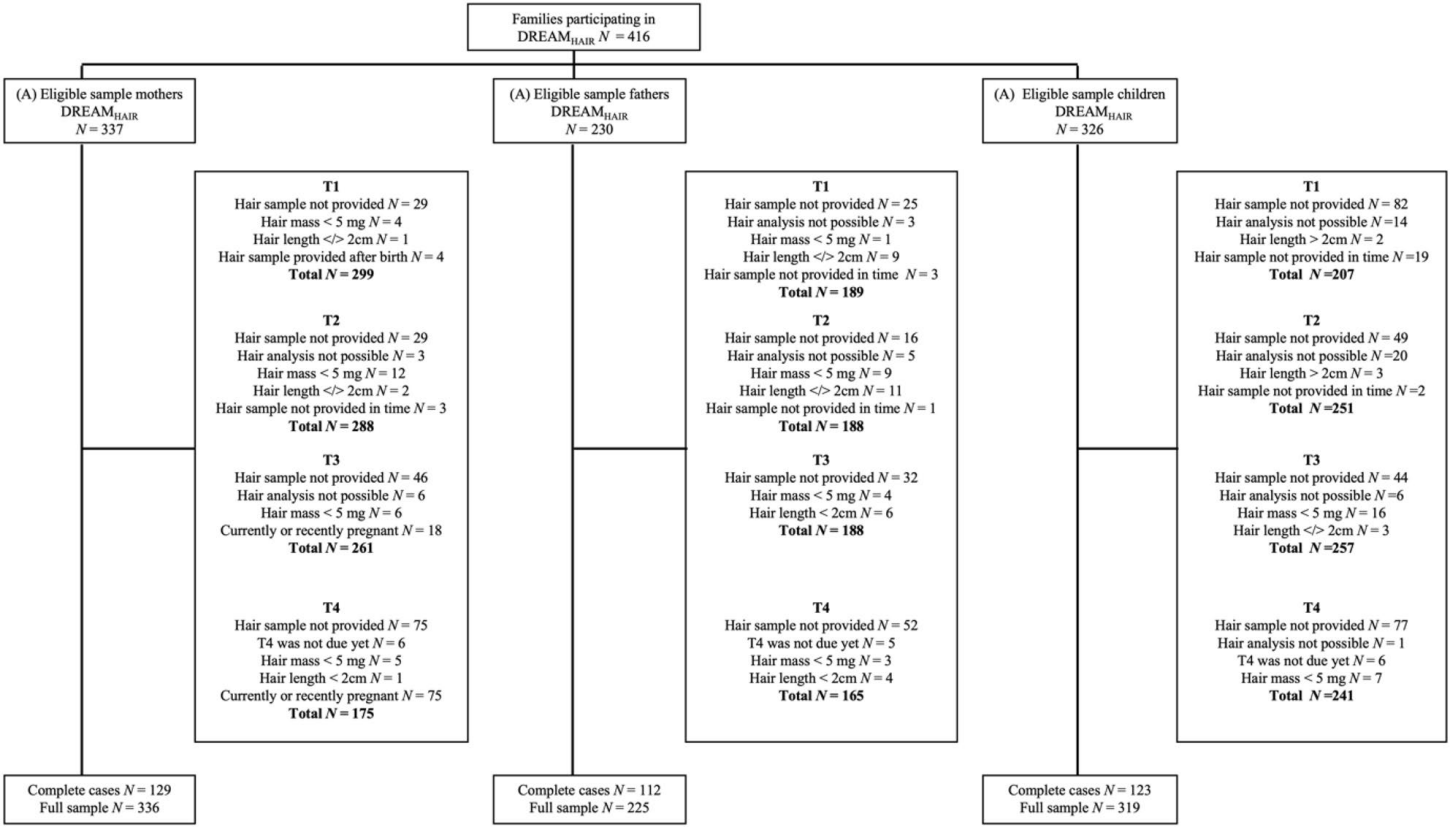
Flowchart of Retention Rate and Exclusion Criteria Resulting in Final Sample. *Note.* Data until end of February 2023 (version 10 of the quality-assured data files, prospective data collection ongoing). Basic inclusion criteria for the DREAM study were currently expecting a child, residing in Dresden (Germany), and adequate German language skills. DREAM_HAIR_-specific inclusion criteria were no hair loss or baldness, a minimal hair length of 2 cm, no use of glucocorticoid containing medication in the last three months, and no serious disease in the last five years (e.g., cancer). For individual timepoints, mothers’, fathers’, and children’s hair samples were excluded if: they did not provide hair samples, analysis of sample was not possible, T4 was not due yet, hair mass of analyzed segment < 5 mg (relevant for children only at T3 and T4), hair length of analyzed segment < 2 cm (not applicable to children at T1 and T2 due to infant hair length frequently being lower at this time) or > 2 cm (relevant for mothers, fathers, and children only at T1 and T2 due to these time points being known for time-sensitive physiological changes during pregnancy, birth, and early postpartum), hair samples were provided too late, or they were pregnant or recently pregnant (relevant only for mothers at T3 and T4).

Recruitment of expectant parents in the Dresden area began in June 2017 and ended in December 2020. T1 DREAM was completed during pregnancy and participants were recruited for DREAM_HAIR_ via a telephone screening if they completed the T1 DREAM questionnaires at least 4 weeks prior to their anticipated birth date. Informed consent was obtained from all participants and for the children from their legal guardians. DREAM_HAIR_-specific questionnaires and hair samples were taken 4 ± 2 weeks before the anticipated birth date for T1, for T2 approximately 8 weeks after the anticipated birth date, for T3 at 14 months after birth, and for T4 at 24 months after birth (see Fig. 1). Hair samples of children were collected at the same time as parental samples, except for T1, which took place approximately 10 days after birth. The current investigation used data from T1 DREAM for sociodemographic information (i.e., age, academic degree, and parity) as well as T1 DREAM_HAIR_ through T4 DREAM_HAIR_ for hair samples and hair-related information of mothers (*N* = 336), fathers (*N* = 225), and children (*N* = 319) (see Fig. 2 for inclusion criteria).

### Hair endocannabinoid and *N*-acylethanolamines analyses

Hair strands (length ≥ 2 cm; collective diameter 3 mm with exception of child hair samples at T1 and T2) were taken scalp-near from a posterior vertex position either by study employees at T1 or by participants themselves after having been provided with instructive materials (i.e., checklist, video link). Samples were stored at room temperature in aluminum foil in dry and dark surroundings until sent to the Kirschbaum laboratory at the Technische Universität Dresden in five batches. AEA, 1-AG/2-AG, SEA, PEA, and OEA were quantified in the scalp-near 2 cm segment following a validated protocol^14^, assumed to reflect EC/NAE secretion of two months before hair sampling. As 2-AG isomerizes rapidly to 1-AG in biological matrices, a sum of the two was used, which is consistent with the fact that both isomers activate CB1 receptors^57^. Also, as analysis in different batches as well as storage time and stressful life circumstances have been shown to influence hair biomarkers^8,58^, their effects were controlled for in absolute stability analyses (see Statistical analyses). Quality control measures of the laboratory verified that the coefficient of variability (CV%) for a salivary cortisol measurement (7.2%), averaged across the five batches, remained below the recommended threshold of 10%.

### Statistical analyses

Analyses were conducted using IBM® SPSS® version 29 and R version 4.2.2^59^. The analyses of this specific investigation were preregistered, while the study itself was not (10.17605/OSF.IO/TC276). Statistical significance was determined using a critical *p*-value of 0.05. Correlational analysis *p*-values were FDR-corrected for multiple testing^60^. Due to their significantly positively skewed distribution, hair ECs/NAEs were log-transformed to reduce skewness. Apart from visualisations and descriptive statistics, all further analyses were conducted with log-transformed data. Extreme outliers in ECs and ECRs were defined as ± 3 *SD*s from the mean using log-transformed data. Extreme outliers were replaced with the highest and lowest measured value within ± 3 *SDs* at each time point for each sample respectively^61^ and non-detectable values, extremely low values below the detection limit^62^, were replaced with the lowest measurable value for each EC/NAE at each measurement point divided by two^63^ (AEA = 0.5–15.2%; PEA = 0.5%; OEA = 0.9%).

This study examined stability, i.e., the extent to which measurements are similar within an individual and across time, via three indicators. First, multiple-test consistency was examined via the intraclass correlation coefficient (*ICC*) as an index reflecting how much of the variance in a variable (i.e., hair EC/NAE concentrations) is attributable to constant mean differences between individuals, such that a high *ICC* indicated high similarity between measurements of the same person, thereby reflecting consistency across multiple tests (*ICC*: < 0.4 poor, 0.4–0.59 fair, 0.6–0.74 good, > 0.75 excellent consistency^64^). The *ICC* was calculated from a fixed linear time random intercept model from SPSS MIXED as this analysis can handle missing data using restricted maximum likelihood (REML^65^). This represents the ICC for a single measurement *(ICC*_*SINGLE*_*)*, i.e., reliability if a single hair sample was used, which is most suitable to how hair analyses are conducted. To compare multiple-test consistency with results from prior investigations, we also calculated multiple-test consistency for the mean value *(ICC*_*MEAN*_*)*, i.e., reliability when the average of all four hair samples is used, employing SPSS RELIABILITY using a two-way mixed-effects model assessing consistency^66^. For mothers and fathers, multiple-test consistency analyses were conducted using measurement for all four time points (T1–T4) and were repeated to include only T3–T4 to contrast stability levels with/without the major life event of pregnancy and childbirth. Secondly, test–retest stability was calculated as the Spearman correlation between an individual’s hair EC/NAE measurements at two time points, reflecting relative stability across measurement occasions in mothers, fathers, and children respectively (*r* < 0.30 (weak), *r* = 0.3–0.59 (moderate), *r* > 0.59 (high) relative stability^6^). See supplements for Pearson correlation results. Finally, we assessed absolute stability, providing information as to whether the average hair EC/NAE concentration across all participants changes significantly across time. A linear mixed model (LMM) was specified for each of the five hair ECs and NAEs for mothers, fathers, and the children separately. The LMM included a random intercept for participants, time points as repeated levels, a fixed effect of time, (*p* > 0.05 for time as a predictor indicating absolute stability^6^), and controlled for fixed effects of batch, storage time, and subjective extraordinary burden experienced in the past three months (yes/no). See supplementary materials for descriptive statistics for batch and storage time. Post-hoc tests with Sidak correction were conducted. As assumptions (i.e., normality and homoscedasticity of residuals) of LMM for AEA were only met when excluding non-detectable values from the analyses, results for hair AEA are reported without imputed non-detectable values and if results differed this is mentioned (see supplementary materials for results with non-detectable values). To explore the relationship between family members’ hair ECs/NAEs across the perinatal period, Spearman correlations were estimated between child and parental hair concentrations at each measurement occasion (see supplements for Pearson correlation results).

## Results

### Descriptive statistics

Table 1 shows descriptive statistics for the three samples. Most mothers were primiparous and about 66% of mothers and fathers had an academic degree. Amongst the children, 53% were female and 47% male. Overall, the range of raw EC/NAE values was rather large, indicating a wide physiological range of EC/NAE concentrations in hair. Welch’s independent samples t-test showed significant differences between maternal and paternal average hair ECs/NAEs across the perinatal period: maternal hair AEA was higher at T2 and T4, paternal 1-AG/2-AG levels were higher at T1, T2, and T3, maternal SEA levels were higher at T4, maternal PEA levels were higher at all measurement points, and maternal OEA levels were higher at T1 and T2.

**Table 1:** Demographic, hair-related, and raw hair EC/NAE concentrations of the study participants.

Variables	Sample of Mothers (*N* = *336)*				Sample of Fathers (*N* = *225)*				Sample of Children (*N* = *319)*			
Time points	T_1_ (*N* = 299)	T_2_ (*N* = 288)	T_3_ (*N* = 261)	T_4_ (*N* = 175)	T_1_ (*N* = 189)	T_2_ (*N* = 188)	T_3_ (*N* = 188)	T_4_ (*N* = 165)	T_1_ (*N* = 207)	T_2_ (*N* = 252)	T_3_ (*N* = 257)	T_4_ (*N* = 241)
***Demographic characteristics***												
Academic degree (*n, %)*	222 (66.7)				146 (66.1)							
Primiparous *(n, %)*	285 (84.8)											
Age in ^a^years, ^b^weeks, ^c^months, *M (SD)*	30.30^a^ (3.93)				32.64^a^ (5.10)				0.98^b^ (0.81)	8.48^b^ (1.40)	13.86^c^ (0.57)	23.90^c^ (0.62)
Medication intake (*n*, %)												
Antibiotics	5 (1.7)	14 (4.9)	9 (3.4)	4 (2.3)	6 (3.2)	2 (1.1)	7 (3.7)	2 (1.2)	5 (2.4)	6 (2.4)	13 (5.1)	6 (2.5)
Glucocorticoid-containing medication	5 (1.7)	1 (0.3)	3 (1.1)	0 (0.0)	5 (2.6)	2 (1.1)	1 (0.5)	1 (0.6)	0 (0.0)	0 (0.0)	3 (1.2)	3 (1.2)
Antidepressant medication	2 (0.7)	2 (0.7)	2 (0.8)	1 (0.6)	1 (0.5)	2 (1.1)	2 (1.1)	2 (1.2)	N/A	N/A	N/A	N/A
Oral contraceptives	0 (0.0)	28 (9.8)	43 (16.7)	37 (21.6)	N/A	N/A	N/A	N/A	N/A	N/A	N/A	N/A
***Hair endocannabinoids and N-acylethanolamines***												
AEA, *M (SD)*	1.24 (1.80)	1.51 (2.52)	2.09 (2.10)	3.05 (2.64)	1.05 (0.96)	1.08 (1.21)	1.56 (1.17)	1.89 (1.41)	2.50 (1.86)	2.07 (2.09)	0.85 (0.71)	0.92 (0.66)
Two-sided Welch’s t-test: hair ECs/NAEs mothers vs. fathers^d^	*p* = .076	***p* = .013**	*p* = .783	***p* < .001**	*p* = .076	***p* = .013**	*p* = .783	***p* < .001**				
2-AG/1-AG, *M (SD)*	42.02 (31.47)	44.14 (32.84)	66.85 (46.56)	82.06 (46.36)	62.56 (48.75)	62.02 (44.71)	74.64 (46.17)	78.08 (36.04)	131.93 (126.54)	294.82 (362.45)	93.01 (62.85)	103.03 (52.1)
Two-sided Welch’s t-test: hair ECs/NAEs mothers vs. fathers^d^	***p* < .001**	***p* < .001**	***p* = .031**	*p* = .831	***p* < .001**	***p* < .001**	***p* = .031**	*p* = .831				
SEA, *M (SD)*	1052.93 (1036.48)	1079.55 (1370.02)	981.45 (1067.06)	792.53 (797.16)	880.51 (693.93)	843.11 (664.92)	760.59 (532.49)	601.00 (340.57)	305.40 (272.61)	872.85 (775.69)	1054.87 (650.10)	761.45 505.93)
Two-sided Welch’s t-test: hair ECs/NAEs mothers vs. fathers^d^	*p* = .102	*p* = .495	*p* = .070	***p* = .040**	*p* = .102	*p* = .495	*p* = .070	***p* = .040**				
PEA, *M (SD)*	3811.10 (4892.80)	3912.25 (5348.57)	2567.28 (3034.85)	2279.98 (2701.83)	2695.28 (2533.96)	2322.33 (1943.53)	1912.17 (1787.80)	1465.37 (905.57)	507.01 (529.43)	1666.02 (1819.07)	1537.88 (1049.43)	1124.45 (700.52)
Two-sided Welch’s t-test: hair ECs/NAEs mothers vs. fathers^d^	***p* = .002**	***p* < .001**	***p* = .022**	***p* = .007**	***p* = .002**	***p* < .001**	***p* = .022**	***p* = .007**				
OEA, *M (SD)*	3233.87 (4866.80)	3543.21 (6200.86)	2242.16 (1145.52)	2278.43 (3975.58)	2147.11 (2560.64)	1738.90 (1844.36)	1560.20 (1776.55)	1291.93 (1030.98)	399.42 (593.53)	1384.87 (2055.98)	1674.42 (1326.20)	1347.11 (942.53)
Two-sided Welch’s t-test: hair ECs/NAEs mothers vs. fathers^d^	***p* = .007**	***p* = .017**	*p* = .160	*p* = .092	***p* = .007**	***p* = .017**	*p* = .160	*p* = .092				

^a^In years. ^b^ in weeks, ^c^ in months. ^d^Two-sided Welch’s independent samples t-test comparing log-transformed hair ECs/NAEs between mothers and fathers at all time points.

### Stability of hair ECs/NAEs

#### Multiple-test consistency

Regarding mothers, multiple-test consistency of the single measure calculated in LMM (Table 2) was poor for ECs/NAEs T1 to T4 (*ICC*_*SINGLE*_ = .20–.35) and improved slightly for T3–T4 for all ECs/NAEs except AEA (*ICC*_*SINGLE*_ = .00–.43). The mean measure evidenced fair to good multiple-test consistency T1–T4 (*ICC*_*MEAN*_ = .51–.73).

**Table 2:** Multiple test consistency of hair ECs/NAEs.

	Mothers			Fathers			Children		
	Single measure	Mean measure		Single measure	Mean measure		Single measure	Mean measure	
	ICC_SINGLE_	ICC_MEAN_	95% CI	ICC_SINGLE_	ICC_MEAN_	95% CI	ICC_SINGLE_	ICC_MEAN_	95% CI
From T1 to T4	(*n* = 336)	(*n* = 129)		(*n* = 225)	(*n* = 112)		*(n* = 319)	*(n* = 123)	
AEA	.20	.51	[.36, .64]	.16	.41	[.21, .57]	.08	.24	[−.01, .44]
2-AG/1-AG	.30	.62	[.50, .72]	.27	.59	[.45, .70]	.04	.15	[−.12, .38]
SEA	.32	.69	[.59, .77]	.30	.58	[.44, .70]	.08	.09	[−.20, .33]
PEA	.35	.71	[.62, .78]	.36	.68	[.57, .77]	.13	.22	[−.03, .42]
OEA	.35	.73	[.64, .80]	.36	.69	[.59, .78]	.19	.36	[.15, .53]
From T3 to T4	(*n* = 278)	(*n* = 158)		*(n* = 202)	*(n* = 151)				
AEA	.00	−.03	[−.41, .25]	.11	.18	[−.13, .40]			
2-AG/1-AG	.39	.58	[.42, .69]	.38	.55	[.38, .68]			
SEA	.42	.60	[.45, .71]	.42	.60	[.44, .71]			
PEA	.43	.59	[.44, .70]	.45	.63	[.49, .73]			
OEA	.37	.55	[.38, .67]	.45	.63	[.49, .73]			

ICC = Intraclass Correlation Coefficient. ICC_SINGLE_ = Single Measures Intraclass Correlation Coefficient. ICC_MEAN_ = Mean Measures Intraclass Correlation Coefficient. 95% CI = 95% Confidence Interval. LMM = Linear mixed model.

Regarding fathers, multiple-test consistency of the single measure was poor for all ECs/NAEs from T1 to T4 (*ICC*_*SINGLE*_ = .16–.36) and improved slightly for all ECs/NAEs apart from AEA for T3–T4 (*ICC*_*SINGLE*_ = 0.11–0.45). The mean measure showed fair to good multiple-test consistency for all ECs/NAEs T1–T4 (*ICC*_*MEAN*_ = 0.41–0.69).

Children showed poor multiple-test consistency from T1 to T4 for all ECs/NAEs in the single (*ICC*_*SINGLE*_ = 0.04–0.19) and mean (*ICC*_*MEAN*_ = 0.09–0.36) measure.

#### Relative stability

For mothers, associations between hair EC/NAE levels across time points were strongest T1–T2. Weak to moderate relative stability was found regarding maternal hair NAE assessments across all measurement occasions. Regarding AEA and 1-AG/2-AG, significant weak to moderate associations were present across most time points, except for associations at T1–T4 and T2–T4 and only regarding AEA for T3–T4 (Table 3 for full results).

**Table 3:** Test–retest relative stability of hair endocannabinoids and *N*-acylethanolamines for mothers, fathers, and children.

		T1–T2	T1–T3	T1–T4	T2–T3	T2–T4	T3–T4
		*r*_*s*_	*r*_*s*_	*r*_*s*_	*r*_*s*_	*r*_*s*_	*r*_*s*_
Mothers	AEA	.56^***^	.30^***^	−.13	.35^***^	−.06	−.01
	2-AG/1-AG	.53^***^	.15^*^	.07	.26^***^	.14	.52^***^
	SEA	.53^***^	.18^**^	.38^***^	.20^**^	.23^**^	.37^***^
	PEA	.52^***^	.24^***^	.33^***^	.29^***^	.19^*^	.31^***^
	OEA	.56^***^	.27^***^	.36^***^	.33^***^	.23^**^	.36^***^
		(*n* = 258)	(*n* = 234)	(*n* = 158)	(*n* = 233)	(*n* = 155)	(*n* = 158)
Fathers	AEA	.51^***^	.35^***^	−.14	.38^***^	−.02	.09
	2-AG/1-AG	.59^***^	.11	.17	.05	.19^*^	.39^***^
	SEA	.64^***^	.13	.20^*^	.17^*^	.16	.42^***^
	PEA	.63^***^	.15	.28^**^	.21^*^	.18^*^	.48^***^
	OEA	.62^***^	.11^***^	.27^***^	.27^***^	.27^***^	.44^***^
		(*n* = 155)	(*n* = 160)	(*n* = 137)	(*n* = 164)	(*n* = 143)	(*n* = 151)
Children	AEA	.31^***^	.11	−.16	.20^**^	−.24^**^	.18^*^
	2-AG/1-AG	.41^***^	−.22^**^	−.14	−.05	−.18*	.24^**^
	SEA	.49^***^	.07	−.18^*^	−.09	−.25^**^	.23^**^
	PEA	.51^***^	.06	−.01	−.05	.05	.26^***^
	OEA	.56^***^	.08	.01	−.02	.15	.14
		(*n* = 182)	(*n* = 169)	(*n* = 158)	(*n* = 203)	(*n* = 185)	(*n* = 210)

*p < .05, ** *p* < .01, *** *p* < .001, FDR-corrected *p*-values. *r*_*s*_ = Spearman rho correlation coefficient.

Fathers showed moderate to high relative stability for T1–T2 for hair ECs/NAEs. Regarding the NAEs PEA and OEA, weak to moderate relative stability was present across time points except T1–T3 for PEA. AEA showed relative stability only at T1–T2, T1–T3, and T2–T3, and 1-AG/2-AG and SEA both showed relative stability at T1–T2 and T3–T4, but not consistently at other times.

Regarding children, weak to moderate relative stability of hair ECs/NAEs was present for all at T1–T2 and for all except OEA at T3–T4. Otherwise, children’s hair ECs/NAEs were inconsistently and sometimes negatively associated with one another across time (Table 3).

#### Absolute stability

**Figure 3:**
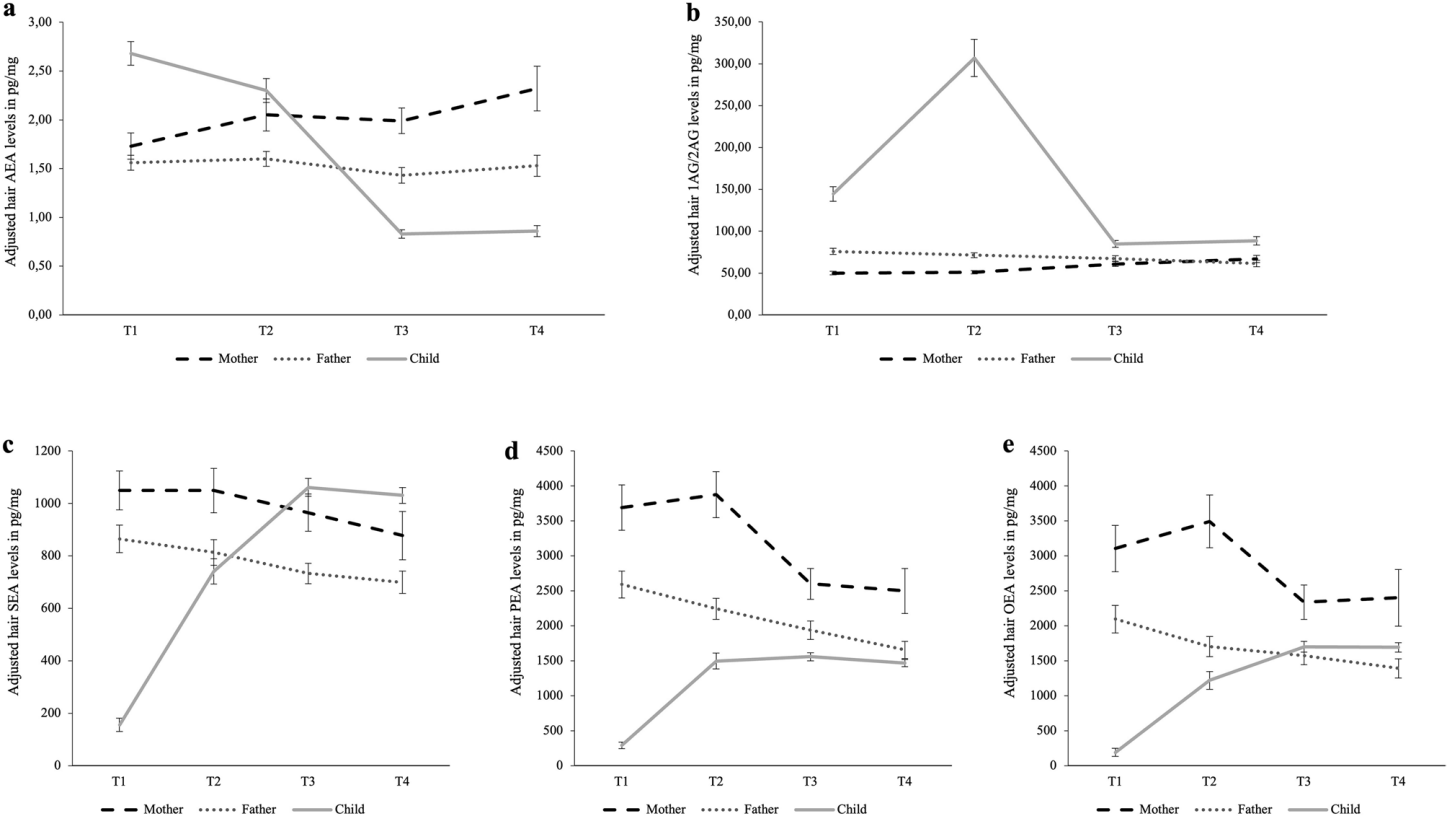
Course of adjusted average raw hair endocannabinoid (AEA, 1-AG/2-AG) and *N*-acylethanolamine (SEA, PEA, OEA) levels from T1 to T4 for mothers, fathers, and children with standard error bars. Estimated marginal means holding all other covariates (batch, storage time, extraordinary burden) at their mean, and standard error bars. AEA results are based on analyses excluding non-detectable values. (**a**) AEA; (**b**) 1-AG/2-AG; (**c**) SEA; (**d**) PEA; (**e**) OEA.

Random intercept LMM adjusting for batch, storage time, and extraordinary burden were conducted to test the effect of time on hair EC/NAEs (see Fig. 3).

In mothers, time (T1–T4) was a significant predictor of maternal AEA (*F* (3, 422.29) = 4.91, *p* = 0.002), 1-AG/2-AG (*F* (3, 477.72) = 4.33, *p* = 0.005), and PEA (*F* (3, 447.24) = 3.08, *p* = 0.027), but not SEA and OEA (all *p* > 0.121). Post-hoc tests showed significant differences between T1–T2 (AEA), T1–T3 (1-AG/2-AG, PEA), T1–T4 (AEA), T2–T3 (1-AG/2-AG, PEA) and T2–T4 (1-AG/2-AG), with earlier assessments showing lower levels of the ECs and higher levels of PEA (Table 4 for pairwise comparisons).

**Table 4:** Pairwise comparisons of hair EC/NAE levels with Sidak correction for mothers, fathers, and children.

	AEA^a b^		1-AG/2-AG^c^		SEA^c^		PEA^c^		OEA^c^	
	*M*_DIFF_	*p*	*M*_DIFF_	*p*	*M*_DIFF_	*p*	*M*_DIFF_	*p*	*M*_DIFF_	*p*
**Mothers**										
T1–T2	−.**09**	**.003**^d^	.00	1.00	.04	.474	.01	1.00	.03	.958
T1–T3	−.08	.115	−.**08**	**.017**	.02	.986	**.10**	**.032**	.11	.104
T1–T4	−.**12**	**.022**	−.09	.098	.02	.996	.10	.282	.09	.618
T2–T3	.01	.999	−.**08**	**.005**	−.02	.983	**.09**	**.038**	.08	.382
T2–T4	−.03	.956^e^	−.**09**	**.043**	−.02	.997	.09	.295	.06	.886
T3–T4	−.04	.601^e^	.01	1.00	−.00	1.00	−.00	1.00	−.02	1.00
**Fathers**										
T1–T2	−.02	.995	.01	.989	.03	.875	.03	.777	.05	.440
T1–T3	.00	1.00	.03	.937	.04	.717	.08	.085	.06	.559
T1–T4	−.01	1.00	.05	.728	.04	.886	.09	.114	.06	.845
T2–T3	.02	.997	.01	.999	.01	.996	.05	.467	.01	1.00
T2–T4	.01	1.00	.03	.905	.01	.999	.06	.420	.00	1.00
T3–T4	−.01	1.00	.02	.934	.00	1.00	.01	.993	−.00	1.00
**Children**										
T1–T2	**.11**	** < .001**	−.**29**	** < .001**	−.**47**	** < .001**	−.**57**	** < .001**	−.**60**	** < .001**
T1–T3	**.51**	** < .001**	**.21**	** < .001**	−.**63**	** < .001**	−.**63**	** < .001**	−.**81**	** < .001**
T1–T4	**.43**	** < .001**	**.18**	** < .001**	−.**64**	** < .001**	−.**61**	** < .001**	−.**82**	** < .001**
T2–T3	**.40**	** < .001**	**.50**	** < .001**	−.**16**	** < .001**	−.06	.071	−.**21**	** < .001**
T2–T4	**.32**	** < .001**	**.48**	** < .001**	−.**17**	** < .001**	−.04	.680	−.**22**	** < .001**
T3–T4	−.07	.082	−.03	.747	−.01	.977	.02	.641	−.00	1.000

*M*_DIFF_ = Mean difference. Numbers in bold = *p* < .05. ^a^Mothers *n* = 331, fathers *n* = 225, children *n* = 315. ^b^Calculated when excluding non-detectable values. ^c^Mothers: *n* = 336, fathers *n* = 225, children *n* = 318. ^d^This comparison was not significant when including non-detectable values. ^e^This comparison was significant when including non-detectable values.

Regarding fathers, time (T1–T4) did not significantly predict hair EC/NAE levels (*p’s* > 0.093; Table 4 for pairwise comparisons).

Regarding children, time (T1–T4) was a significant predictor of all hair ECs/NAEs (AEA: *F* (3, 417.79) = 89.77, *p* < 0.001; 1-AG/2-AG: *F* (3, 479.05) = 97.53, *p* < 0.001; SEA: *F* (3, 509.27) = 185.90, *p* < 0.001; PEA: *F* (3, 529.53) = 129.71, *p* < 0.001; OEA: *F* (3, 495.29) = 127.25, *p* < 0.001). Post-hoc tests showed that hair EC/NAE levels differed significantly between all time points (*p* < 0.001), except between T3 and T4 (all ECs/NAEs: *p* > 0.086), between T2 and T3 (PEA: *p* = 0.071), and between T2 and T4 (PEA: *p* = 0.680; Table 4). Results were the same when calculated separately for child sex, apart from no significant AEA level difference T1–T2 for female children (*p* = 0.065; supplementary materials).

### Parent–child associations in hair ECs/NAEs across the perinatal period

Within mother–child and father-child dyads, all ECs and NAEs showed statistically significant small-to-medium positive correlations at almost all measurement points, apart from 1-AG/2-AG at T4 in mother–child dyads and AEA and OEA at T1 in father-child dyads (Table 5). The strength of correlations was highest at T4 for mother–child dyads (except for 1-AG/2-AG), whereas for father-child dyads this was the case at T3.

**Table 5:** Parent–child and couple associations of hair endocannabinoids and *N*-acylethanolamines.

		T1	T2	T3	T4
		*r*_*s*_	*r*_*s*_	*r*_*s*_	*r*_*s*_
Mother–child^a^	AEA	.28***	.46***	.25***	.44***
	2-AG/1-AG	.39***	.40***	.19*	.07
	SEA	.37***	.24**	.25**	.25**
	PEA	.28***	.24***	.29***	.30***
	OEA	.27***	.21**	.20**	.31***
Father-child^b^	AEA	.14	.37***	.37***	.33***
	2-AG/1-AG	.40***	.41***	.46***	.23*
	SEA	.39***	.36***	.45***	.37***
	PEA	.24*	.38***	.50***	.45***
	OEA	.21	.36***	.44***	.37***
Mother–father^c^	AEA	.29***	.42***	.48***	.42***
	2-AG/1-AG	.51***	.46***	.52***	.27*
	SEA	.30***	.32***	.38***	.28*
	PEA	.24**	.26**	.42***	.31**
	OEA	.18*	.26**	.37***	.26*

*p < .05, ** *p* < .01, *** *p* < .001, FDR-corrected *p*-values. *r*_*s*_ = Spearman correlation coefficient.

^a^T1 *n* = 183; T2 *n* = 214–215; T3 *n* = 212; T4 *n* = 153.

^b^T1 *n* = 117; T2 *n* = 141–142; T3 *n* = 154; T4 *n* = 146.

^c^T1 *n* = 167; T2 *n* = 160; T3 *n* = 153; T4 *n* = 98.

Within couples, ECs/NAEs again were statistically significantly positively correlated at all measurement occasions (with the exception of AEA and OEA at T1 and T4) in the small to medium range. Strength of inter-parental associations seemed to increase from T1, reaching a peak at T3 and lower values at T4.

## Discussion

This prospective investigation examined whether a core assumption of hair EC/NAE analysis, namely that hair EC/NAE concentrations are largely stable within an individual, also holds true in the perinatal period in mothers, fathers, and their children and also when experiencing the major life event of pregnancy and childbirth in mothers and fathers. As a secondary aim, we investigated how hair ECs/NAEs are associated within the family unit across the first two years after birth.

### Descriptive classification of hair EC/NAE levels across the perinatal period

In general, the physiological range of hair ECs/NAEs varied widely in both adults and children, consistent with prior research on ECs/NAEs in serum^67^ and hair^32,45^. Regarding mothers, hair AEA concentrations were descriptively higher than in studies with women within^45^ and outside the perinatal period^27,32^. In contrast, average 1-AG/2-AG levels were lower at T1, and increased at T4 to a level similar to women outside the perinatal period^32^. NAE levels at T1 and T3 corresponded to previous research with postpartum mothers^45,50^ and were higher than in women outside the perinatal period^32^, but decreased to similar levels by T4. Fathers’ hair AEA and 1-AG/2-AG across all time points were descriptively higher than reported outside the perinatal period^14^, whereas hair NAE concentrations were elevated at T1, but decreased to levels similar to outside the perinatal period by T4^14^. Regarding children, concentrations can be discussed in the context of Hitzler et al.’s^45^ study, where they assessed hair ECs/NAEs in mothers and children shortly after birth and at one year postpartum. Shortly after birth, hair 1-AG/2-AG and SEA levels were descriptively lower and hair PEA and OEA higher than in Hitzler et al.’s^45^ investigation, whereas all hair ECs/NAEs were higher in this study at 14 months after birth than the levels in Hitzler et al.’s^45^ study at twelve months. Overall, relating findings of hair EC/NAE levels between different studies is hampered by differences in hair segment length being analyzed, variations due to continuously evolving laboratory techniques, and different hair sample storage times.

### Stability of parental hair ECs/NAEs across the perinatal period

Regarding the stability of hair ECs/NAEs of mothers and fathers across the perinatal period (T1–T4), this study found poor multiple-test consistency regarding all ECs/NAEs on the single measure. Our finding suggests that, when using a single hair sample, between 16% and 36% of the variance in hair ECs/NAEs across the perinatal period in mothers and fathers may be attributed to between-person differences. Thus 64% to 84% of the variance in a single hair sample is attributable to within-person differences, so dependent on for instance situational factors. The intraindividual stable part is considerably lower than the 59% to 82% found for HCC^5^, which could be explained by the fact that EC synthesis is predominantly “on demand” in response to specific signals^18^. It should be noted that multiple-test consistency was lowest for AEA in the present study and that mean measures consistency (i.e., consistency when the average of several hair samples is used rather than a single hair sample) of hair ECs/NAEs with a range between 41% and 73% was overall lower compared to Gao et al.’s^32^ investigation of 100 women outside the perinatal period (range 79% to 92%). This may be attributable to unique stressors and changes present during the perinatal period, although even when examining only the later postpartum period (T3 and T4) was consistency lower than reported previously.

Regarding the effect of the major life event of pregnancy and childbirth on stability of parental hair ECs/NAEs, findings showed that the single measure ICC was slightly lower when including pregnancy and childbirth for both mothers (2–10%) and fathers (9–12%) for all hair ECs/NAEs except hair AEA. This finding indicates that consistency of a single hair sample taken in the later postpartum period may reflect slightly more stable components than when taken at any time in the perinatal period. Considering the small changes, results suggest that hair ECs/NAEs show minimally less stability in the presence of the major life event of pregnancy and childbirth and are thus similarly useful as a biological marker for parents in the perinatal period. Future studies would benefit from investigating other major life events (e.g., graduation, relocation) to corroborate our finding.

Furthermore, results indicated weak to moderate test–retest relative stability for hair NAEs for mothers and fathers. Regarding AEA and 1-AG/2-AG, test–retest stability was present for T1 to T2 for both parents, but less consistently at later time-points. As the time between T1 and T2 (about 3 months) was shorter than between assessments from T2 onwards (10 to 24 months), this could be a potential factor explaining why associations were particularly strong from T1 to T2 for all hair ECs/NAEs and may indicate that for mothers and fathers AEA and 1-AG/2-AG show short-term stability but may vary more across time compared to NAEs. Our findings partly align with Hitzler et al.^45^, who reported significant associations between maternal hair NAEs assessed shortly after birth and 12 months after birth, but found no significant relations regarding AEA and 1-AG/2-AG. However, Gao et al.^32^ demonstrated moderate-to-high associations across six six-month intervals for all hair ECs/NAEs, suggesting that time period between assessments and the unique circumstances of the perinatal period may influence the strength of associations. Overall, it should also be noted that associations between hair ECs/NAEs shortly after birth (T2) with those after 14 months were weaker for all ECs/NAEs except AEA compared to associations in the later postpartum period (i.e., between 14 and 24 months), which could imply that relative stability increases with greater distance to the major life event of pregnancy and childbirth.

Finally, results showed that across the perinatal period SEA and OEA in mothers and all hair ECs/NAEs in fathers demonstrated absolute stability. This suggests that hair EC/NAE concentrations averaged across all fathers in this study did not change significantly across the perinatal period, while this was not the case for mothers. Given that pregnancy, childbirth, and the early postpartum period entail major physiological changes in mothers^35^, including changes to the ECS and HPA axis^36,68^, it fits that mean levels AEA, 1-AG/2-AG, and PEA were less stable for mothers than fathers in the present study. Research has shown that a successful pregnancy requires coordinated regulation of EC/NAE levels in reproductive system tissues^68^ and altered HPA-axis functioning with increasing tonic glucocorticoid secretion^38,69^. Specifically, maternal hair AEA during the third pregnancy trimester was significantly lower than at 8 weeks and at two years after birth, partly aligning with findings by Kumbholz et al.^36^ who also found an increase from third trimester to 9 weeks after birth. 1-AG/2-AG during pregnancy and 8 weeks after birth was significantly lower than at 14 months, consistent with prior research showing maternal 1-AG/2-AG increases from pregnancy to 12 months after birth^45^. Finally, PEA levels were higher during pregnancy and 8 weeks after birth than at 14 months, which was descriptively also the case in Hitzler et al.^45^, albeit not significant. Results indicate that regulation of the maternal ECS may change during pregnancy and postpartum, with potential implications for glucocorticoid regulation and immune system functioning in this time. Both AEA and 1-AG/2-AG have been characterized as HPA axis regulators^70^, with AEA correlating negatively with cortisol^27,28,36^, and 2-AG being attributed the role of terminating the HPA response^71^, with their increasing levels postpartum potentially supporting a restoration of pre-pregnancy HPA-axis functioning. Moreover, PEA has been attributed anti-inflammatory, analgesic, and neuroprotective properties^72^. Thus, changes in these ECs/NAEs may reflect adaptive responses to regain homeostasis in the context of cortisol increases over pregnancy^73^ and an inflammation-immunosuppression-inflammation phenotype during pregnancy^74^. It remains for future research to disentangle the exact timeline of EC/NAE secretion patterns across the perinatal period to understand their contribution and interplay.

### Stability of child hair ECs/NAEs across the perinatal period

Results further demonstrated poor multiple-test consistency for child hair ECs/NAEs taken shortly after birth up to two years of age, indicating a low stable component in this time (4–19%), and no absolute stability. Furthermore, findings indicated that while all hair ECs/NAEs of children were significantly positively correlated in the early postpartum period (T1–T2), relative stability was subsequently only consistently present from 14 to 24 months after birth for AEA, 1-AG/2-AG, SEA, and PEA. This pattern could indicate that the first two years of life may represent a particularly sensitive and dynamic developmental period for the ECS^75^ during which marked ECS changes occur. This is further underscored by negative associations of child hair ECs/NAEs at T1 or T2 with those at T3 or T4. This may indicate that higher levels in infancy may be associated with reduced levels in toddlerhood and vice versa. It should also be considered that during pregnancy the maternal and fetal ECS interact via the fetoplacental unit^76^, such that at T1 and T2 child EC/NAE levels may be more strongly influenced by the respective intrauterine milieu, whereas at later time points, extrauterine influences may dominate. Furthermore, maternal EC lipids, mainly 2-AG, may be transferred to the child during breastfeeding^77,78^, which could also contribute to why early 1-AG/2-AG levels associate negatively with later levels. Taken together, our findings of low stability for children align with the only other study assessing EC/NAE levels in child hair in the perinatal period up to one year after birth^45^ and low intra-individual stability in salivary cortisol measured in 5 to 8 month old infants^79^.

Similar to Hitzler et al.^45^, children’s hair 1-AG/2-AG and AEA levels decreased from shortly after birth to 14 months. However, in our study 1-AG/2-AG concentrations increased markedly in hair samples taken 8 weeks after birth, reflecting the early postnatal period. Due to the scarcity of investigations in humans, these findings may be carefully discussed in relation to animal research with rats also showing a peak in 2-AG levels in the rat brain during early postnatal development followed by subsequent decreases^80,81^. It has been suggested that elevated 2-AG during early development may function to induce suckling behavior and support (neuro)development and growth^82^. In contrast to our study showing decreases in hair AEA, rat studies found that AEA levels in the brain increase from birth into adulthood^80,83^. Furthermore, research suggests that in neonates and infants there is a greater relative expression of AEA degrading (i.e., FAAH) compared to synthesizing (i.e., NAPE-PLD) enzymes in the human dorsolateral prefrontal cortex, which reduces with increasing age and switches around toddlerhood in favor of AEA synthesizing enzymes^75^. However, comparability is limited as we measured peripheral EC/NAE levels in this study and the ECS is widespread throughout the body, thereby hampering comparisons with region-specific findings^14^.

Regarding child NAEs, findings align with Hitzler et al.^45^ showing increased hair NAE levels at 14 months after birth compared to T1 shortly after birth, where NAE levels were markedly below parental levels. Importantly, sensitivity analyses by child sex indicated no major differences between male and female children regarding hair EC/NAE trajectories up to 24 months after birth, contrasting research indicating sex-divergent ECS functioning due to crosstalk with sex steroid hormones^43,84^. Finally, results showed no significant differences between 14 and 24 months of age in any hair EC/NAE levels in children, possibly indicating that the ECS may gradually become more stable as development progresses, similar to findings regarding HCC^48^. However, further research investigating children’s hair EC/NAE levels across childhood and adolescence is needed to better understand developmental trajectories. This is particularly relevant when considering research showing that the ECS becomes functionally active already during early developmental stages in humans and rodents and its expression patterns in the brain implicates a role also in neurodevelopment^43,44^.

### Inter-family associations of hair ECs/NAEs

Findings indicated that maternal, paternal, and child hair ECs/NAEs were significantly positively cross-sectionally associated with one another across the perinatal period. While these findings contradict previous research in a comparably small sample on ECs/NAEs^45,50^, they are in line with HCC research, where positive associations between all family members at child age 6 years were found^55^ as well as positive maternal-child associations reported at various child ages (9–12 months^52^; 2 years^85^; 4–5 years^86^; 5–14 years^53^; 7–14 years^51^; 14 years^87^; but see^47,54^ for no associations at 9–12 months and shortly after birth, respectively). The observed correspondence may be explained by an interplay of genetic and environmental factors. Associations in the mother–child dyad at T1 could partly reflect intrauterine interactions of the maternal and child ECS via the fetoplacental unit^76^. Research indicates that endocannabinoid receptors (CB1, CB2) and synthesizing and degrading enzymes are expressed in the placenta and that the placenta regulates local endocannabinoid tonus, with implications for correct placental functioning and labor^43,68,76^. Yet, our finding that positive associations persist beyond the intrauterine period and are present also in father-child dyads, even at T1, imply additional mechanisms. The finding of hair EC/NAE mother–child and father-child correspondence suggests a heritable familial trait of the ECS. This fits with recent twin-study research suggesting heritability in endocannabinoid signaling^88^ and showing that in 138 twins (45 monozygotic; 24 dizygotic) serum endocannabinoid ligands had a certain degree of heritability (AEA 46%; OEA 35%; PEA 39%; SEA 34%), but were also markedly influenced by the environment^89^. A role for environmental factors also seems particularly plausible considering we found significant maternal-paternal associations. While positive assortative mating, a preference for individuals similar to oneself, could partly explain these findings^90^, environmental factors including various psychological and lifestyle factors shared or similar within the family context also seem plausible. For instance, nutrition and food intake, stress exposure, sleeping pattern, or physical activity level have all been linked to the ECS^43,71,91,92^ and could contribute to ECS correspondence between family members. Furthermore, considering transfer of maternal EC lipids, particularly 2-AG, to the child via breast milk has been suggested^77,78^, maternal-child correspondence in the postpartum period could partly be attributable to breastfeeding practices. However, the fact that we found paternal-child associations of similar or greater strength in the postpartum period suggests that genetics and other shared environmental influences are equally important. Considering this study covered the first 24 months after birth, contact intensity and frequency likely declines with time hereafter and future research should examine trajectories of inter-family correspondence in hair ECs/NAEs as the child ages to see whether a decline in correspondence can be detected similar to findings with HCC^53^. Taken together, our results carefully suggest hair ECs/NAEs are related between family members across the perinatal period, however more research is needed to tease apart genetic and environmental contributions.

### Strengths and limitations

Key strengths include the prospective design, our large sample size, our focus specifically on the perinatal period, and including all family members. However, limitations exist. Firstly, the operationalization of pregnancy and childbirth as a major life event was done post-hoc and not during the design stage of the study and we were not able to confirm an increase in subjective stress as our study lacked subjective stress assessments such as the widely used Perceived Stress Scale^93^. Moreover, as the present investigation is longitudinal, batch effects present a complication^94^ that may also affect biomarker concentrations in hair samples^8^, which we statistically controlled for in absolute stability analyses. Furthermore, it remains to be determined what peripheral EC/NAE levels measured in hair samples exactly reflect in terms of ECS functioning, as a first investigation found no significant link with blood and urine EC/NAE levels^95^. Also, while this study focused specifically on examining stability and inter-family relations of hair ECs/NAEs across the perinatal period, future investigations should examine in detail which psychological factors (e.g., traumatic experiences, depressive and anxiety symptoms) could explain variance in hair EC/NAE levels in this time. Finally, it should be noted that the present sample involved a community with above-average health and education status^56^. Hence, investigations in more heterogeneous samples are needed to evaluate generalizability of findings.

## Conclusion

Overall, the present investigation revealed that hair ECs/NAEs show between 16% and 36% stability across the perinatal period in mothers and fathers, with only minor reductions in the presence of a major life event (i.e., pregnancy and childbirth). However, only fathers showed consistent absolute stability in this time, whereas the ECS of mothers undergoes significant changes during pregnancy and early postpartum. Our findings also suggest hair ECs/NAEs are not stable across time within children in the first two years of life, indicating a developmental window. These discoveries provide important insights into the analysis of hair ECs/NAEs, indicating that this method, while evidencing lower stability than hypothesised, could be well-suited for research applications seeking to measure EC/NAE secretion changes in adults and children. Finally, this study highlights for the first time the family as a unit that may exert significant effects on ECS functioning in the period from late pregnancy to two years after birth, thus highlighting the need for future research to further examine how this relates to parental psychological and child developmental outcomes.

### Supplementary Information

Supplementary Information.

## Data Availability

The datasets analysed during the current study are not publicly available due legal and ethical constraints (i.e., public sharing of participant data was not included in the informed consent of the study) but are available from the corresponding author on reasonable request.

## References

[1] Greff MJE (2019). Hair cortisol analysis: An update on methodological considerations and clinical applications. Clin. Biochem..

[2] Stalder T, Kirschbaum C (2012). Analysis of cortisol in hair – State of the art and future directions. Brain. Behav. Immun..

[3] Russell E, Koren G, Rieder M, Van Uum S (2012). Hair cortisol as a biological marker of chronic stress: Current status, future directions and unanswered questions. Psychoneuroendocrinology.

[4] Chen Z (2019). Determination, intercorrelation and intraindividual stability of five steroids in hair, saliva and urine among chinese college students. Steroids.

[5] Stalder T (2012). Intraindividual stability of hair cortisol concentrations. Psychoneuroendocrinology.

[6] Zhang Q, Chen Z, Chen S, Xu Y, Deng H (2017). Intraindividual stability of cortisol and cortisone and the ratio of cortisol to cortisone in saliva, urine and hair. Steroids.

[7] Liu CH, Snidman N, Leonard A, Meyer J, Tronick E (2016). Intra-individual stability and developmental change in hair cortisol among postpartum mothers and infants: Implications for understanding chronic stress. Dev. Psychobiol..

[8] Marceau K, Rolan E, Robertson OC, Wang W, Shirtcliff EA (2021). Within-person changes of cortisol, dehydroepiandrosterone, testosterone, estradiol, and progesterone in hair across pregnancy, with comparison to a non-pregnant reference group. Compr. Psychoneuroendocrinology.

[9] Hillard CJ (2018). Circulating endocannabinoids: From whence do they come and where are they going?. Neuropsychopharmacology.

[10] Hauer D (2013). Plasma concentrations of endocannabinoids and related primary fatty acid amides in patients with post-traumatic stress disorder. PLoS ONE.

[11] Hill MN (2013). Reductions in circulating endocannabinoid levels in individuals with post-traumatic stress disorder following exposure to the world trade center attacks. Psychoneuroendocrinology.

[12] Schaefer C (2014). Fatty acid ethanolamide levels are altered in borderline personality and complex posttraumatic stress disorders. Eur. Arch. Psychiatry Clin. Neurosci..

[13] Voegel CD, Baumgartner MR, Kraemer T, Wüst S, Binz TM (2021). Simultaneous quantification of steroid hormones and endocannabinoids (ECs) in human hair using an automated supported liquid extraction (SLE) and LC-MS/MS – Insights into EC baseline values and correlation to steroid concentrations. Talanta.

[14] Gao W, Walther A, Wekenborg M, Penz M, Kirschbaum C (2020). Determination of endocannabinoids and N-acylethanolamines in human hair with LC-MS/MS and their relation to symptoms of depression, burnout, and anxiety. Talanta.

[15] Skaper SD, Di Marzo V (2012). Endocannabinoids in nervous system health and disease: the big picture in a nutshell. Philos. Trans. R. Soc. B Biol. Sci..

[16] Walker OS, Holloway AC, Raha S (2019). The role of the endocannabinoid system in female reproductive tissues. J. Ovarian Res..

[17] Micale V, Drago F (2018). Endocannabinoid system, stress and HPA axis. Eur. J. Pharmacol..

[18] Lu H-C, Mackie K (2021). Review of the endocannabinoid system. Biol. Psychiatry Cogn. Neurosci. Neuroimaging.

[19] Devane WA (1992). Isolation and structure of a brain constituent that binds to the cannabinoid receptor. Science.

[20] Sugiura T (1995). 2-Arachidonoylgylcerol: A possible endogenous cannabinoid receptor ligand in brain. Biochem. Biophys. Res. Commun..

[21] Hauer, D., Toth, R. & Schelling, G. Endocannabinoids, “New-Old” Mediators of Stress Homeostasis. In *Stress Challenges and Immunity in Space* (ed. Choukèr, A.) 181–204 (Springer, Cham, 2020). 10.1007/978-3-030-16996-1_10.

[22] Tsuboi K, Uyama T, Okamoto Y, Ueda N (2018). Endocannabinoids and related N-acylethanolamines: biological activities and metabolism. Inflamm. Regen..

[23] Stalder T (2017). Stress-related and basic determinants of hair cortisol in humans: A meta-analysis. Psychoneuroendocrinology.

[24] Henderson GL (1993). Mechanisms of drug incorporation into hair. Forensic Sci. Int..

[25] Pragst F, Balikova MA (2006). State of the art in hair analysis for detection of drug and alcohol abuse. Clin. Chim. Acta.

[26] Thieme, D., Anielski, P., Helfers, A.-K. & Krumbholz, A. Analytical approaches to the quantitative evaluation of endocannabinoids and glucocorticoids as stress markers: Growing evidence for hair testing. In *Stress Challenges and Immunity in Space* (Springer, Cham, 2020).

[27] Walther A (2023). Depressive symptoms are negatively associated with hair N-arachidonoylethanolamine (anandamide) levels: A cross-lagged panel analysis of four annual assessment waves examining hair endocannabinoids and cortisol. Prog. Neuropsychopharmacol. Biol. Psychiatry.

[28] Bergunde L (2023). Childbirth-related posttraumatic stress symptoms – examining associations with hair endocannabinoid concentrations during pregnancy and lifetime trauma. Transl. Psychiatry.

[29] Wilker S (2016). Endocannabinoid concentrations in hair are associated with PTSD symptom severity. Psychoneuroendocrinology.

[30] Voegel CD (2022). Alterations of stress-related glucocorticoids and endocannabinoids in hair of chronic cocaine users. Int. J. Neuropsychopharmacol..

[31] Tam FI (2021). Hair endocannabinoid concentrations in individuals with acute and weight-recovered anorexia nervosa. Prog. Neuropsychopharmacol. Biol. Psychiatry.

[32] Gao W, Schmidt K, Enge S, Kirschbaum C (2021). Intra-individual stability of hair endocannabinoid and N-acylethanolamine concentrations. Psychoneuroendocrinology.

[33] Luhmann M, Hofmann W, Eid M, Lucas RE (2012). Subjective well-being and adaptation to life events: A meta-analysis. J. Pers. Soc. Psychol..

[34] Horsch, A. & Ayers, S. Childbirth and stress. In *Stress: Concepts, Cognition, Emotion, and Behavior* 325–330 (Elsevier, 2016). 10.1016/B978-0-12-800951-2.00040-6.

[35] Tan EK, Tan EL (2013). Alterations in physiology and anatomy during pregnancy. Best Pract. Res. Clin. Obstet. Gynaecol..

[36] Krumbholz A, Anielski P, Reisch N, Schelling G, Thieme D (2013). Diagnostic value of concentration profiles of glucocorticosteroids and endocannabinoids in hair. Ther. Drug Monit..

[37] Duthie L, Reynolds RM (2013). Changes in the maternal hypothalamic-pituitary-adrenal axis in pregnancy and postpartum: Influences on maternal and fetal outcomes. Neuroendocrinology.

[38] King LS, Humphreys KL, Cole DA, Gotlib IH (2022). Hair cortisol concentration across the peripartum period: Documenting changes and associations with depressive symptoms and recent adversity. Compr. Psychoneuroendocrinology.

[39] Berg SJ, Wynne-Edwards KE (2001). Changes in testosterone, cortisol, and estradiol levels in men becoming fathers. Mayo Clin. Proc..

[40] Edelstein RS (2015). Prenatal hormones in first-time expectant parents: Longitudinal changes and within-couple correlations: Prenatal Hormones in First-Time Expectant Parents. Am. J. Hum. Biol..

[41] Gettler LT, McDade TW, Feranil AB, Kuzawa CW (2011). Longitudinal evidence that fatherhood decreases testosterone in human males. Proc. Natl. Acad. Sci..

[42] Geller P (2004). Pregnancy as a stressful life event. CNS spectrums.

[43] Almeida MM, Dias-Rocha CP, Calviño C, Trevenzoli IH (2022). Lipid endocannabinoids in energy metabolism, stress and developmental programming. Mol. Cell. Endocrinol..

[44] Galve-Roperh I, Palazuelos J, Aguado T, Guzmán M (2009). The endocannabinoid system and the regulation of neural development: potential implications in psychiatric disorders. Eur. Arch. Psychiatry Clin. Neurosci..

[45] Hitzler M (2023). Longitudinal course of endocannabinoids and *N* -acylethanolamines in hair of mothers and their children in the first year postpartum: investigating the relevance of maternal childhood maltreatment experiences. Psychol. Med..

[46] Gray NA (2018). Determinants of hair cortisol concentration in children: A systematic review. Psychoneuroendocrinology.

[47] Liu CH, Snidman N, Leonard A, Meyer J, Tronick E (2016). Intra-individual stability and developmental change in hair cortisol among postpartum mothers and infants: Implications for understanding chronic stress: Hair Cortisol Stability in Mothers and Infants. Dev. Psychobiol..

[48] Karlén J, Frostell A, Theodorsson E, Faresjö T, Ludvigsson J (2013). Maternal influence on child HPA axis: A prospective study of cortisol levels in hair. Pediatrics.

[49] Tollenaar MS, Jansen J, Beijers R, Riksen-Walraven JM, de Weerth C (2010). Cortisol in the first year of life: Normative values and intra-individual variability. Early Hum. Dev..

[50] Koenig AM (2018). Altered hair endocannabinoid levels in mothers with childhood maltreatment and their newborns. Biol. Psychol..

[51] Hollenbach JP (2019). Hair cortisol, perceived stress, and social support in mother–child dyads living in an urban neighborhood. Stress.

[52] Liu CH, Fink G, Brentani H, Brentani A (2017). An assessment of hair cortisol among postpartum Brazilian mothers and infants from a high-risk community in São Paulo: Intra-individual stability and association in mother-infant dyads. Dev. Psychobiol..

[53] Perry NB, Donzella B, Troy MF, Barnes AJ (2022). Mother and child hair cortisol during the COVID-19 pandemic: Associations among physiological stress, pandemic-related behaviors, and child emotional-behavioral health. Psychoneuroendocrinology.

[54] Karl M (2023). The association between maternal symptoms of depression and hair glucocorticoids in infants across the perinatal period. Psychoneuroendocrinology.

[55] Dauegaard S, Olsen NJ, Heitmann BL, Larsen SC (2020). Familial associations in hair cortisol concentration: A cross-sectional analysis based on the Healthy Start study. Psychoneuroendocrinology.

[56] Kress V (2019). The impact of parental role distributions, work participation, and stress factors on family health-related outcomes: Study protocol of the prospective multi-method cohort “dresden study on parenting, work, and mental health” (DREAM). Front. Psychol..

[57] Vogeser M, Schelling G (2007). Pitfalls in measuring the endocannabinoid 2-arachidonoyl glycerol in biological samples. Clin. Chem. Lab. Med..

[58] Steudte-Schmiedgen S (2017). Hair cortisol concentrations and cortisol stress reactivity in generalized anxiety disorder, major depression and their comorbidity. J. Psychiatr. Res..

[59] R Core Team. R: A language and environment for statistical computing. R Foundation for Statistical Computing (2022).

[60] Benjamini Y, Hochberg Y (1995). Controlling the false discovery rate: A practical and powerful approach to multiple testing. J. R. Stat. Soc. Ser. B Methodol..

[61] Ghosh, D. & Vogt, A. Outliers: An evaluation of methodologies. *Joint statistical meetings* (2012).

[62] Herbers J (2021). How to deal with non-detectable and outlying values in biomarker research: Best practices and recommendations for univariate imputation approaches. Compr. Psychoneuroendocrinol..

[63] Walther A (2021). Depressive symptoms are not associated with long-term integrated testosterone concentrations in hair. World J. Biol. Psychiatry.

[64] Cicchetti DV (1994). Guidelines, criteria, and rules of thumb for evaluating normed and standardized assessment instruments in psychology. Psychol. Assess..

[65] Bartlett MS (1937). Properties of sufficiency and statistical tests. Proc. R Soc. Lond. Ser. - Math. Phys. Sci..

[66] Koo TK, Li MY (2016). A guideline of selecting and reporting intraclass correlation coefficients for reliability research. J. Chiropr. Med..

[67] Felton SJ (2017). Serum endocannabinoids and N-acyl ethanolamines and the influence of simulated solar UVR exposure in humans in vivo. Photochem. Photobiol. Sci..

[68] Kozakiewicz ML, Grotegut CA, Howlett AC (2021). Endocannabinoid system in pregnancy maintenance and labor: A mini-review. Front. Endocrinol..

[69] Jung C (2011). A longitudinal study of plasma and urinary cortisol in pregnancy and postpartum. J. Clin. Endocrinol. Metab..

[70] deRoon-Cassini TA, Stollenwerk TM, Beatka M, Hillard CJ (2020). Meet your stress management professionals: The endocannabinoids. Trends Mol. Med..

[71] Morena M, Patel S, Bains JS, Hill MN (2016). Neurobiological interactions between stress and the endocannabinoid system. Neuropsychopharmacology.

[72] Mock ED, Gagestein B, van der Stelt M (2023). Anandamide and other N-acylethanolamines: A class of signaling lipids with therapeutic opportunities. Prog. Lipid Res..

[73] Howland MA, Sandman CA, Glynn LM (2017). Developmental origins of the human hypothalamic-pituitary-adrenal axis. Expert Rev. Endocrinol. Metab..

[74] True H, Blanton M, Sureshchandra S, Messaoudi I (2022). Monocytes and macrophages in pregnancy: The good, the bad, and the ugly*. Immunol. Rev..

[75] Long LE, Lind J, Webster M, Weickert CS (2012). Developmental trajectory of the endocannabinoid system in human dorsolateral prefrontal cortex. BMC Neurosci..

[76] Keimpema E, Calvigioni D, Harkany T (2013). Endocannabinoid signals in the developmental programming of delayed-onset neuropsychiatric and metabolic illnesses. Biochem. Soc. Trans..

[77] Fride, E. *et al.* Chapter 6 The Endocannabinoid System During Development: Emphasis on Perinatal Events and Delayed Effects. In *Vitamins & Hormones* vol. 81 139–158 (Elsevier, 2009). 10.1016/S0083-6729(09)81006-6 19647111

[78] Datta P (2021). Human milk endocannabinoid levels as a function of obesity and diurnal rhythm. Nutrients.

[79] De Weerth C, Van Geert P (2002). A longitudinal study of basal cortisol in infants: Intra-individual variability, circadian rhythm and developmental trends. Infant Behav. Dev..

[80] Berrendero F, Sepe N, Ramos JA, Di Marzo V, Fernández -Ruiz, J. J.  (1999). Analysis of cannabinoid receptor binding and mRNA expression and endogenous cannabinoid contents in the developing rat brain during late gestation and early postnatal period. Synapse.

[81] Fride E (2009). The endocannabinoid system during development: emphasis on perinatal events and delayed effects. Vitam. Horm..

[82] Fride E (2001). Critical role of the endogenous cannabinoid system in mouse pup suckling and growth. Eur. J. Pharmacol..

[83] Bukiya, A. N. Physiology of the Endocannabinoid System During Development. In *Recent Advances in Cannabinoid Physiology and Pathology* (ed. Bukiya, A. N.) vol. 1162 13–37 (Springer, Cham, 2019). 10.1007/978-3-030-21737-2_2 31332732

[84] Viveros M (2012). The endocannabinoid system in critical neurodevelopmental periods: sex differences and neuropsychiatric implications. J. Psychopharmacol. (Oxf.).

[85] Bryson HE, Mensah F, Goldfeld S, Price AMH, Giallo R (2021). Hair cortisol in mother-child dyads: Examining the roles of maternal parenting and stress in the context of early childhood adversity. Eur. Child Adolesc. Psychiatry.

[86] Schloß S (2019). Hair cortisol concentration in mothers and their children: roles of maternal sensitivity and child symptoms of attention-deficit/hyperactivity disorder. J. Neural Transm..

[87] Olstad DL (2016). Hair cortisol levels, perceived stress and body mass index in women and children living in socioeconomically disadvantaged neighborhoods: the READI study. Stress.

[88] Koethe D (2019). Familial abnormalities of endocannabinoid signaling in schizophrenia. World J. Biol. Psychiatry.

[89] Bermingham KM (2021). Genetic and environmental influences on serum oxylipins, endocannabinoids, bile acids and steroids. Prostaglandins Leukot. Essent. Fatty Acids.

[90] Robinson MR (2017). Genetic evidence of assortative mating in humans. Nat. Hum. Behav..

[91] Jurado-Fasoli L (2022). Acute and long-term exercise differently modulate plasma levels of oxylipins, endocannabinoids, and their analogues in young sedentary adults: A sub-study and secondary analyses from the ACTIBATE randomized controlled-trial. BioMedicine.

[92] Kesner AJ, Lovinger DM (2020). Cannabinoids, endocannabinoids and sleep. Front. Mol. Neurosci..

[93] Karam F (2012). Reliability and validity of the 4-item perceived stress scale among pregnant women: Results from the OTIS antidepressants study. Res. Nurs. Health.

[94] Leek JT (2010). Tackling the widespread and critical impact of batch effects in high-throughput data. Nat. Rev. Genet..

[95] Barba SV, Kirschbaum C, Gao W (2023). Endocannabinoid and Perceived Stress: Association Analysis of Endocannabinoid Levels in Hair Versus Levels in Plasma and Urine. Biol. Psychol..

