# Supplementary material for: Stability and inter-family associations of hair endocannabinoid and N-acylethanolamines across the perinatal period in mothers, fathers, and children
Source: Sci Rep. 2024 Apr 24;14:9459. doi: 10.1038/s41598-024-59818-6 (PMC11043453; doi:10.1038/s41598-024-59818-6)

Supplementary materials for the manuscript

"Stability and inter-family associations of hair endocannabinoid and N-acyl-ethanolamines across the perinatal period in mothers, fathers, and children"

Table S1

Descriptive statistics for batch and storage time

**Variables**	**Sample of Mothers** (*N = 336)*				**Sample of Fathers** (*N =225)*				**Sample of Children** (*N =319)*			
Time points	T_1 (*N* = 299)	T_2 (*N* = 288)	T_3 (*N* = 261)	T_4 (*N* = 175)	T_1 (*N* = 189)	T_2 (*N* = 188)	T_3 (*N* = 188)	T_4 (*N* = 165)	T_1 (*N* = 207)	T_2 (*N* = 252)	T_3 (*N* = 257)	T_4 (*N* = 241)
Batch *(n, %)*												
Batch 1	80 (26.8)	74 (25.7)	0 (0.0)	0 (0.0)	54 (28.6)	46 (24.5)	0 (0.0)	0 (0.0)	55 (26.6)	56 (22.2)	0 (0.0)	0 (0.0)
Batch 2	130 (43.5)	127 (44.1)	66 (25.3)	0 (0.0)	75 (39.7)	79 (42.0)	48 (25.5)	0 (0.0)	93 (44.9)	117 (46.4)	68 (26.5)	0 (0.0)
Batch 3	73 (24.4)	69 (24.0)	111 (42.5)	53 (30.3)	58 (25.4)	48 (25.5)	70 (37.2)	54 (32.7)	46 (22.2)	62 (24.6)	101 (39.3)	67 (27.8)
Batch 4	12 (4.0)	18 (6.3)	67 (25.7)	73 (41.7)	9 (4.8)	15 (8.0)	55 (29.3)	63 (38.2)	10 (4.8)	17 (6.7)	73 (28.4)	106 (44.0)
Batch 5	4 (1.3)	0 (0.0)	17 (6.5)	49 (28.0)	3 (1.6)	0 (0.0)	15 (8.0)	48 (29.1)	3 (1.4)	0 (0.0)	15 (5.8)	68 (28.2)
Storage time in weeks, *M (SD)*	57.03 (15.90)	44.33 (14.21)	40.38 (13.58)	37.00 (15.28)	56.90 (16.49)	44.50 (14.87)	41.95 (17.09)	38.48 (15.21)	52.08 (16.17)	44.54 (14.11)	39.78 (13.50)	37.37 (15.20)

Table S2

Pairwise comparisons of hair AEA levels with Sidak correction for mothers, fathers, and children when including imputed non-detectable values

	Mothers (n = 336)		Fathers (n = 225)		Children (n = 318)	
**Comparisons**	AEA		AEA		AEA	
	M_{DIFF}	p	M_{DIFF}	p	M_{DIFF}	p
T1–T2	-.11	.054[a]	-.06	.922	**.14**	**< .001**
T1–T3	-.04	.970	-.13	.293	**.80**	**< .001**
T1–T4	**-.28**	**<.001**	-.19	.077	**.67**	**< .001**
T2–T3	.06	.795	-.07	.787	**.66**	**< .001**
T2–T4	**-.18**	**.009[b]**	-.13	.272	**.53**	**< .001**
T3–T4	**-.24**	**<.001[b]**	-.06	.833	-.12	.254

Note. M_{DIFF} = Mean difference. Numbers in bold = $p < .05$. [a] This comparison was significant when excluding non-detectable values. [b] This comparison was not significant when excluding non-detectable values.

Table S3

Pairwise comparisons of hair EC/NAE levels with Sidak correction for female and male children separately

	AEA[c]		1-AG/2-AG		SEA		PEA		OEA	
	M_{DIFF}	*p*	M_{DIFF}	*p*	M_{DIFF}	*p*	M_{DIFF}	*p*	M_{DIFF}	*p*
**Female children[a]**										
T1–T2	.09	.065	**-.36**	**< .001**	**-.45**	**< .001**	**-.56**	**< .001**	**-.63**	**< .001**
T1–T3	**.43**	**< .001**	**.25**	**< .001**	**-.59**	**< .001**	**-.63**	**< .001**	**-.84**	**< .001**
T1–T4	**.35**	**< .001**	**.18**	**.002**	**-.62**	**< .001**	**-.63**	**< .001**	**-.89**	**< .001**
T2–T3	**.34**	**< .001**	**.61**	**< .001**	**-.14**	**< .001**	-.08	.233	**-.21**	**< .001**
T2–T4	**.26**	**< .001**	**.54**	**< .001**	**-.17**	**< .001**	-.07	.376	**-.26**	**< .001**
T3–T4	-.08	.207	-.07	.158	-.02	.891	.00	1.000	-.04	.558
**Male children[b]**										
T1–T2	**.14**	**.007**	**-.25**	**< .001**	**-.46**	**< .001**	**-.57**	**< .001**	**-.56**	**< .001**
T1–T3	**.54**	**< .001**	**.16**	**< .001**	**-.64**	**< .001**	**-.62**	**< .001**	**-.77**	**< .001**
T1–T4	**.55**	**< .001**	**.16**	**.004**	**-.63**	**< .001**	**-.57**	**< .001**	**-.73**	**< .001**
T2–T3	**.41**	**< .001**	**.41**	**< .001**	**-.19**	**< .001**	-.05	.725	**-.21**	**< .001**
T2–T4	**.41**	**< .001**	**.41**	**< .001**	**-.18**	**< .001**	.00	1.000	**-.17**	**.015**
T3–T4	.01	1.000	.00	1.000	-.01	.999	.05	.282	.04	.748

Note. M_{DIFF} = Mean difference. Numbers in bold = $p < .05$. [a] $n = 155$ for AEA, $n = 157$ for 1-AG/2-AG, SEA, PEA, and OEA. [b] $n = 139$ for AEA, $n = 140$ for 1-AG/2-AG, SEA, PEA, and OEA. [c] Calculated when excluding non-detectable values.

Table S4

Test-retest relative stability of hair endocannabinoids and N-acyl-ethanolamines for mothers, fathers, and children calculated using Pearson correlation.

		T1–T2	T1–T3	T1–T4	T2–T3	T2–T4	T3–T4
		r	*r*	*r*	*r*	*r*	*r*
**Mothers**	AEA	.44***	.21*	-.03	.24**	.11	-.02
	2-AG/1-AG	.50***	.16*	.07	.31***	.17	.41***
	SEA	.53***	.20**	.37***	.23**	.19*	.43***
	PEA	.56***	.24***	.32***	.30***	.22*	.42***
	OEA	.54***	.25***	.38***	.36***	.23**	.38***
		(*n*=258)	(*n*=234)	(*n*=158)	(*n*=233)	(*n*=155)	(*n*=158)
**Fathers**	AEA	.48***	.10	-.02	.15	.06	.10
	2-AG/1-AG	.59***	.11	.18	.08	.23*	.39***
	SEA	.63***	.16	.17	.16	.14	.43***
	PEA	.61***	.24**	.32***	.26***	.18	.47***
	OEA	.62***	.18*	.27**	.31***	.25**	.46***
		(*n*=155)	(*n*=160)	(*n*=137)	(*n*=164)	(*n*=143)	(*n*=151)
**Children**	AEA	.23**	.14	-.06	.22**	-.11	.07
	2-AG/1-AG	.41***	-.23**	-.12	-.05	-.14	.20*
	SEA	.44***	.07	-.16	-.11	-.27***	.26***
	PEA	.46***	-.01	-.03	-.08	.01	.23***
	OEA	.52***	.11	.03	.00	.13	.14
		(*n*=182)	(*n*=169)	(*n*=158)	(*n*=203)	(*n*=185)	(*n*=210)

Note. * $p < .05$, ** $p < .01$, *** $p < .001$, FDR-corrected *p*-values. *r* = Pearson correlation coefficient.

Table S5

Parent-child associations of hair endocannabinoids and N-acylethanolamines calculated using Pearson correlation.

		T1	T2	T3	T4
		r	*r*	*r*	*r*
**Mother-Child** [a]	AEA	.20*	.34***	.21**	.39***
	2-AG/1-AG	.40***	.40***	.23**	.04
	SEA	.27***	.22**	.25**	.27**
	PEA	.18*	.18*	.21**	.33**
	OEA	.19*	.18*	.18*	.38***
**Father-Child** [b]	AEA	.02	.20	.33***	.20
	2-AG/1-AG	.40***	.39***	.50***	.24*
	SEA	.33***	.33***	.44***	.35***
	PEA	.26**	.40***	.42***	.44***
	OEA	.21	.39***	.41***	.35***
**Mother-Father** [c]	AEA	.13	.24*	.39***	.19
	2-AG/1-AG	.52***	.44***	.53***	.32**
	SEA	.28***	.29***	.36***	.27*
	PEA	.24**	.28**	.38***	.25*
	OEA	.17*	.27**	.38***	.23*

Notes. * $p < .05$, ** $p < .01$, *** $p < .001$, FDR-corrected *p*-values. *r* = Pearson correlation coefficient.
[a] T1 $n = 183$; T2 $n = 214–215$; T3 $n = 212$; T4 $n = 153$.
[b] T1 $n = 117$; T2 $n = 141–142$; T3 $n = 154$; T4 $n = 146$.
[c] T1 $n = 167$; T2 $n = 160$; T3 $n = 153$; T4 $n = 98$.

Relative Stability Mothers

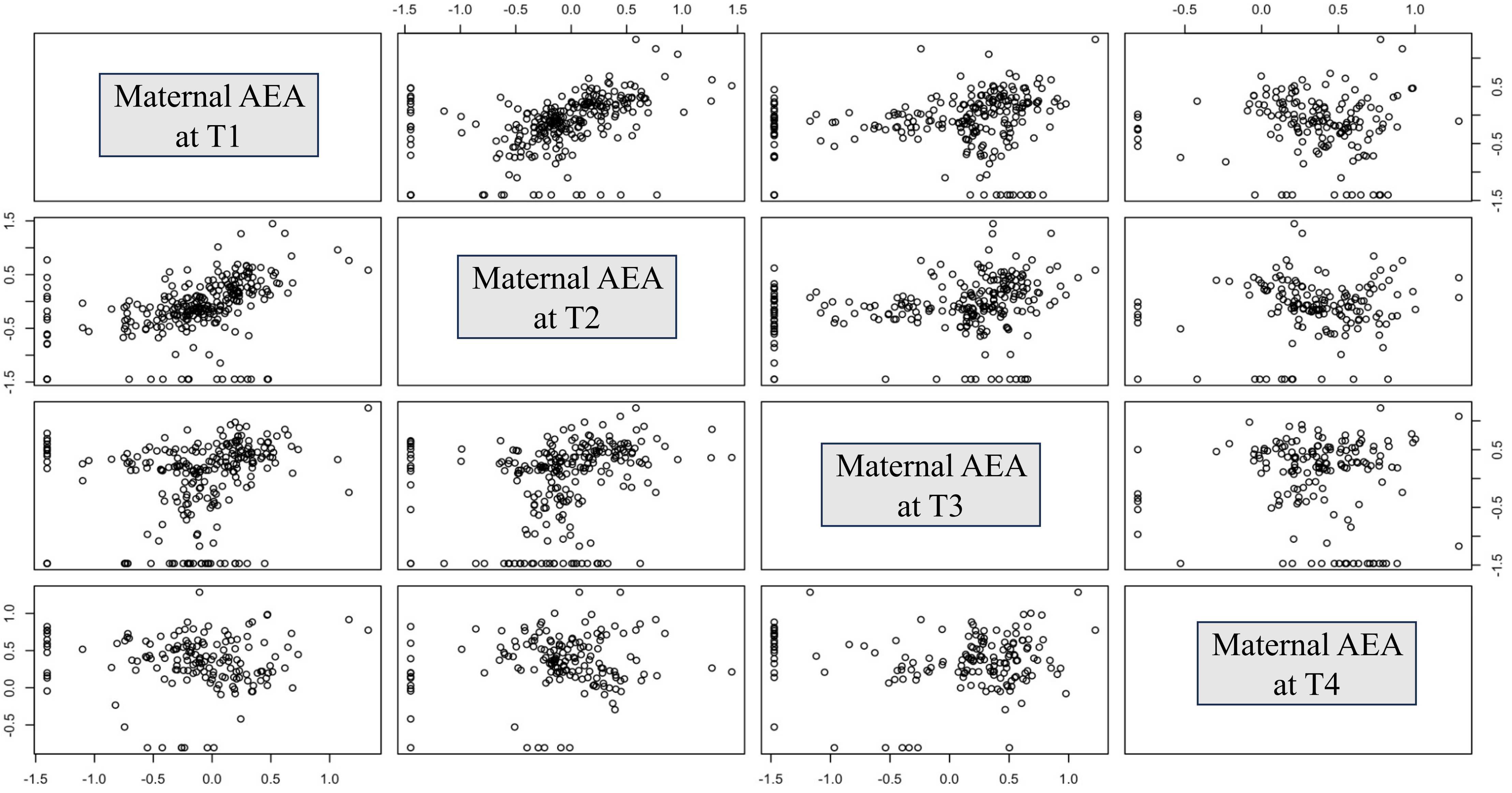

Relative Stability Mothers

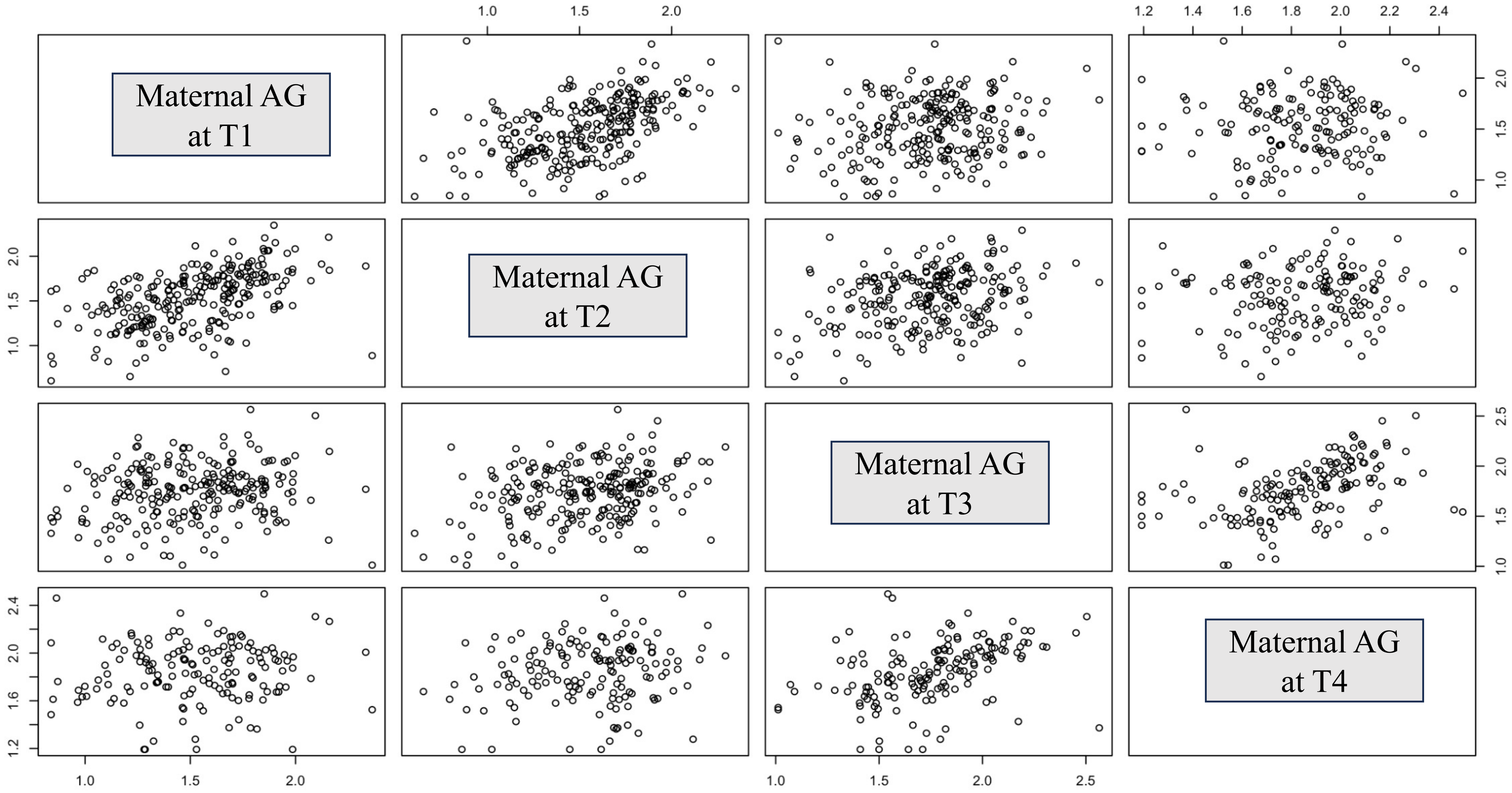

Relative Stability Mothers

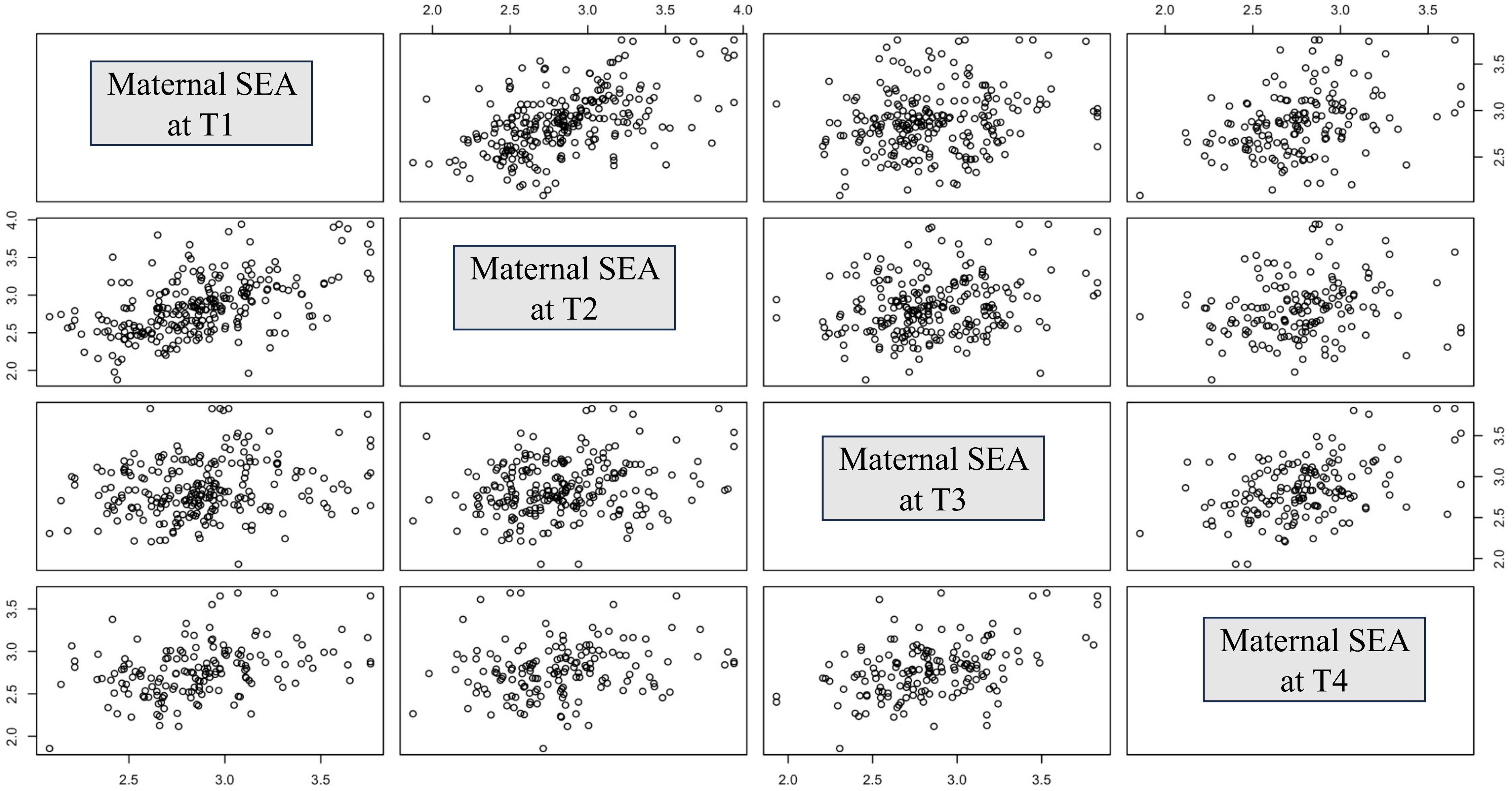

Relative Stability Mothers

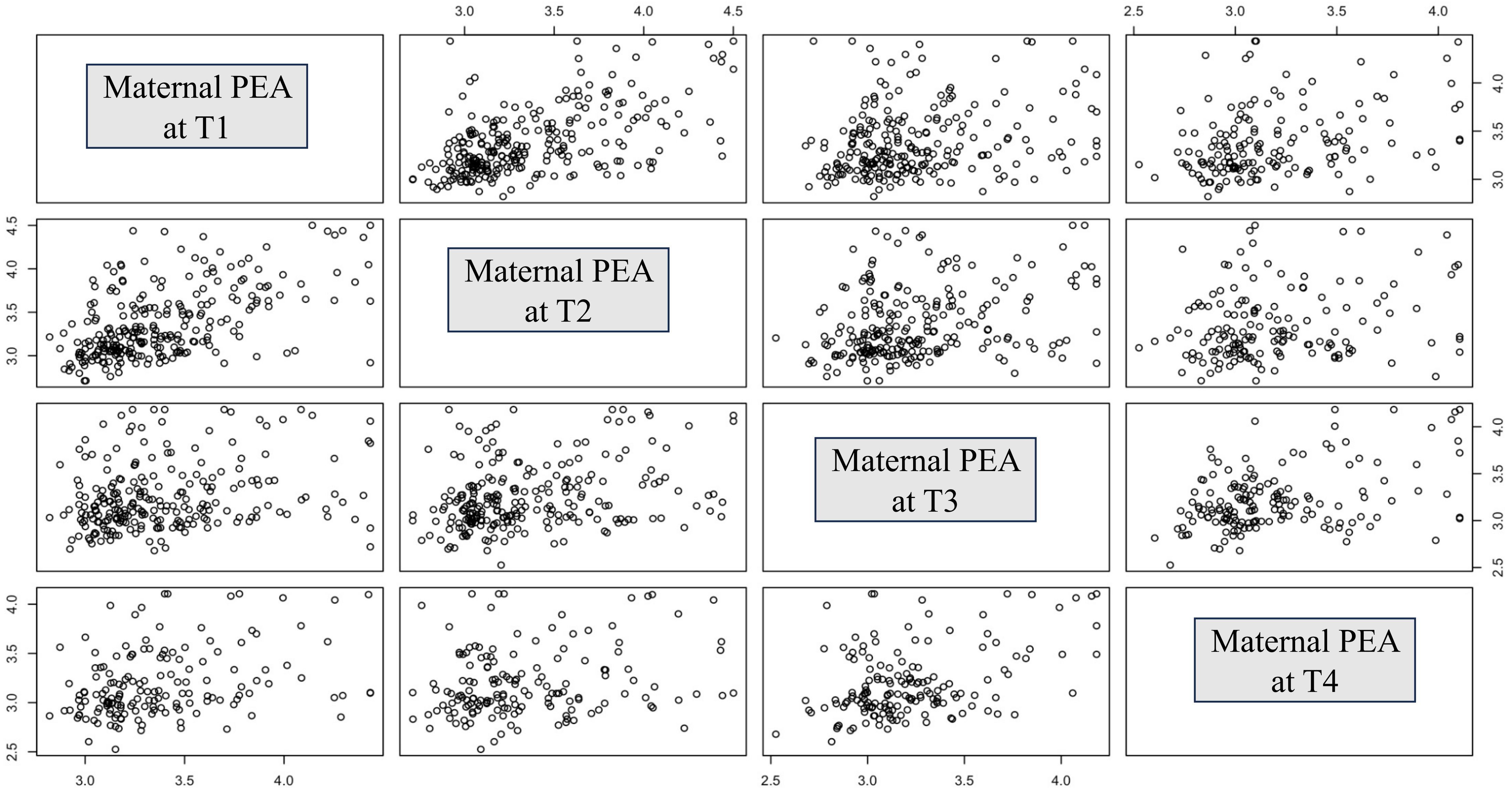

Relative Stability Mothers

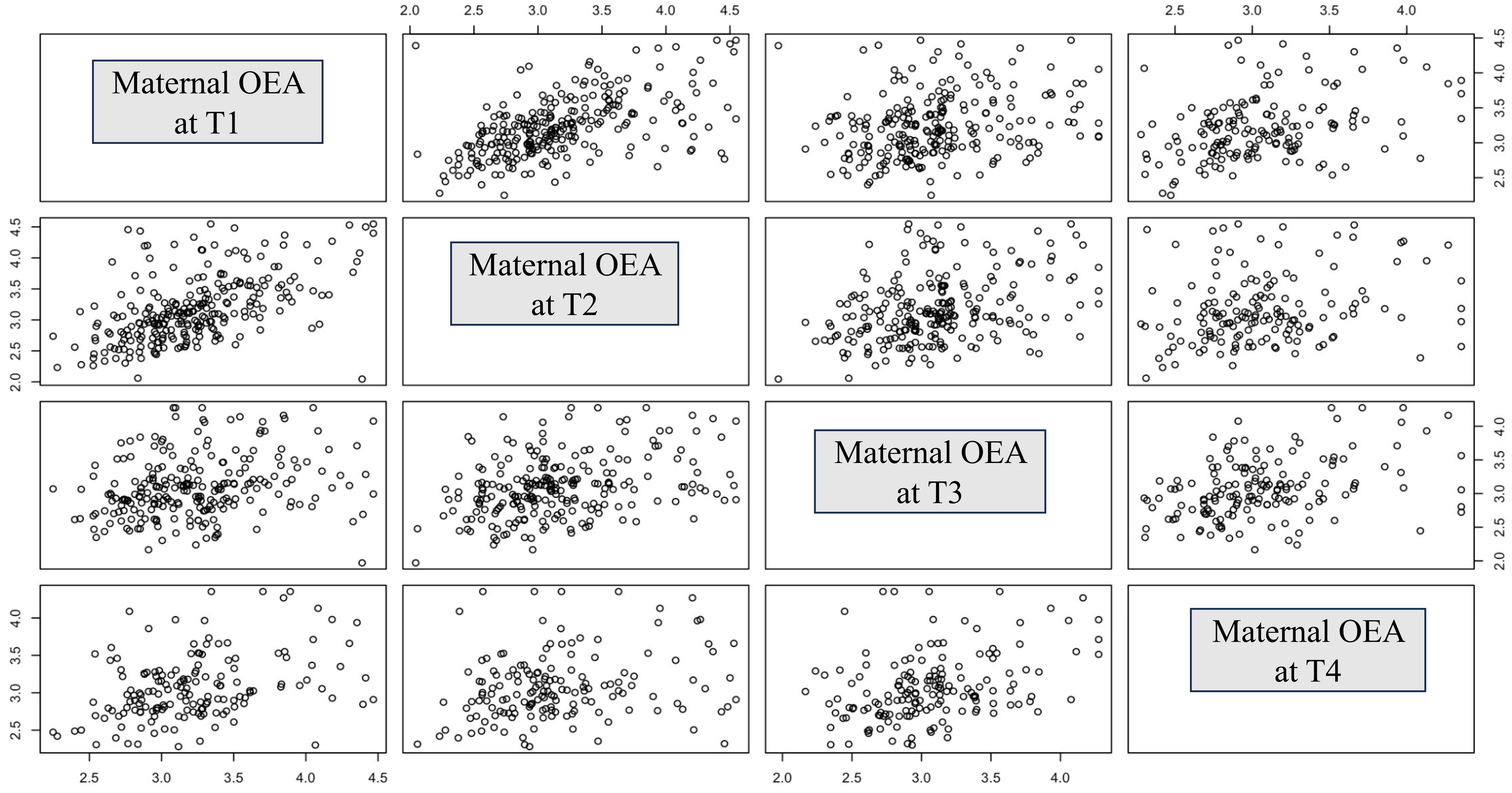

Relative Stability Fathers

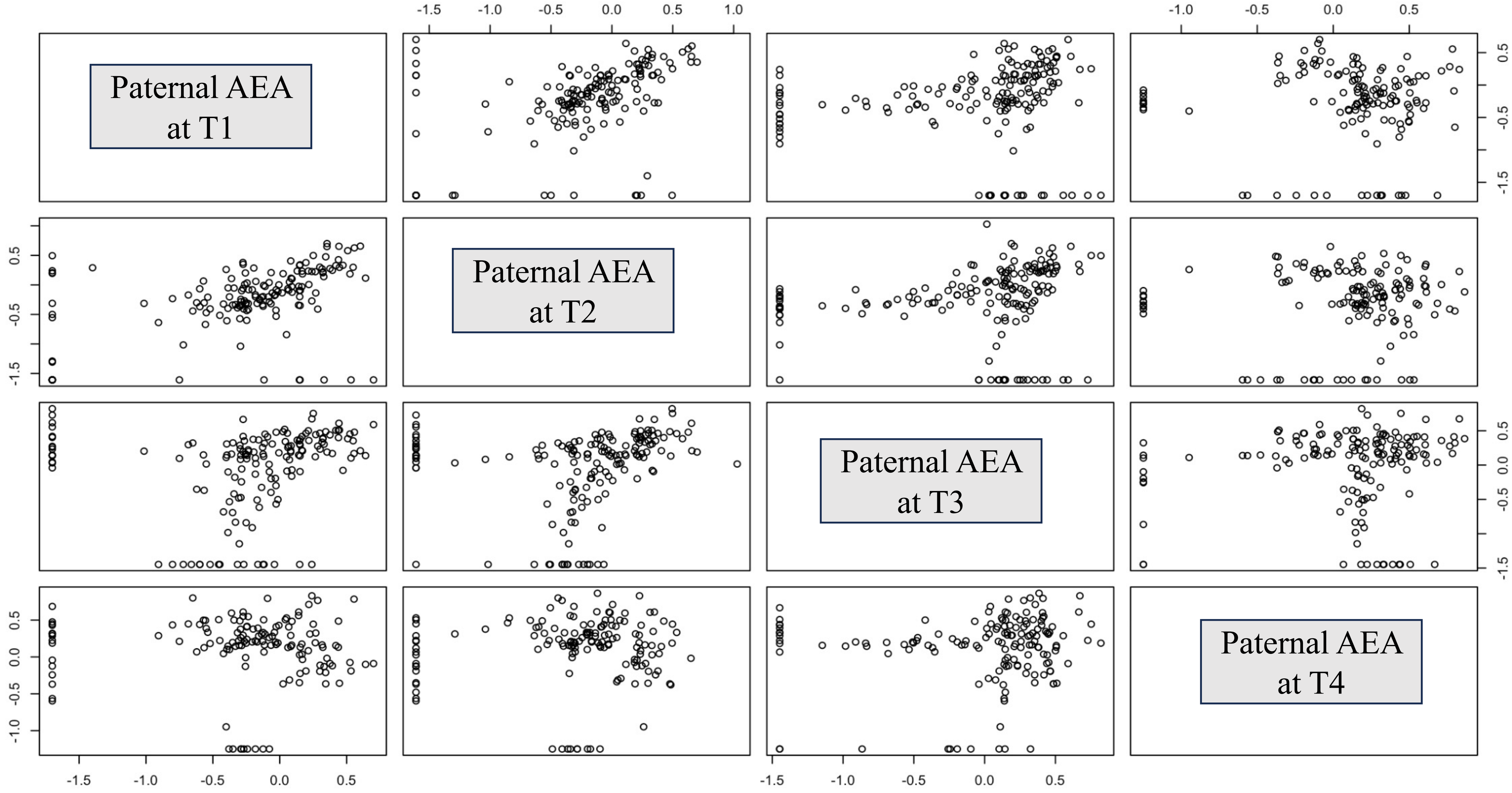

Relative Stability Fathers

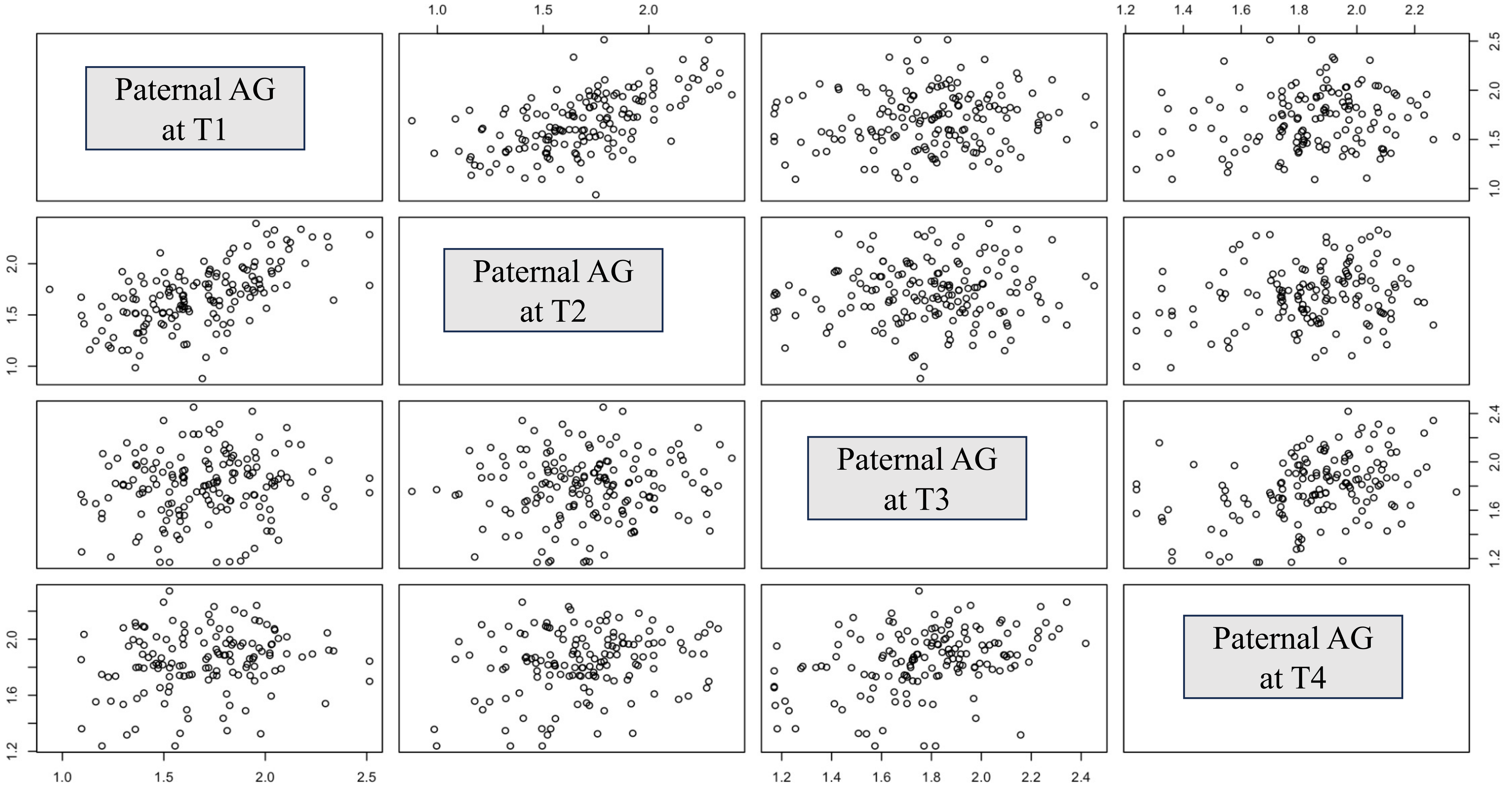

Relative Stability Fathers

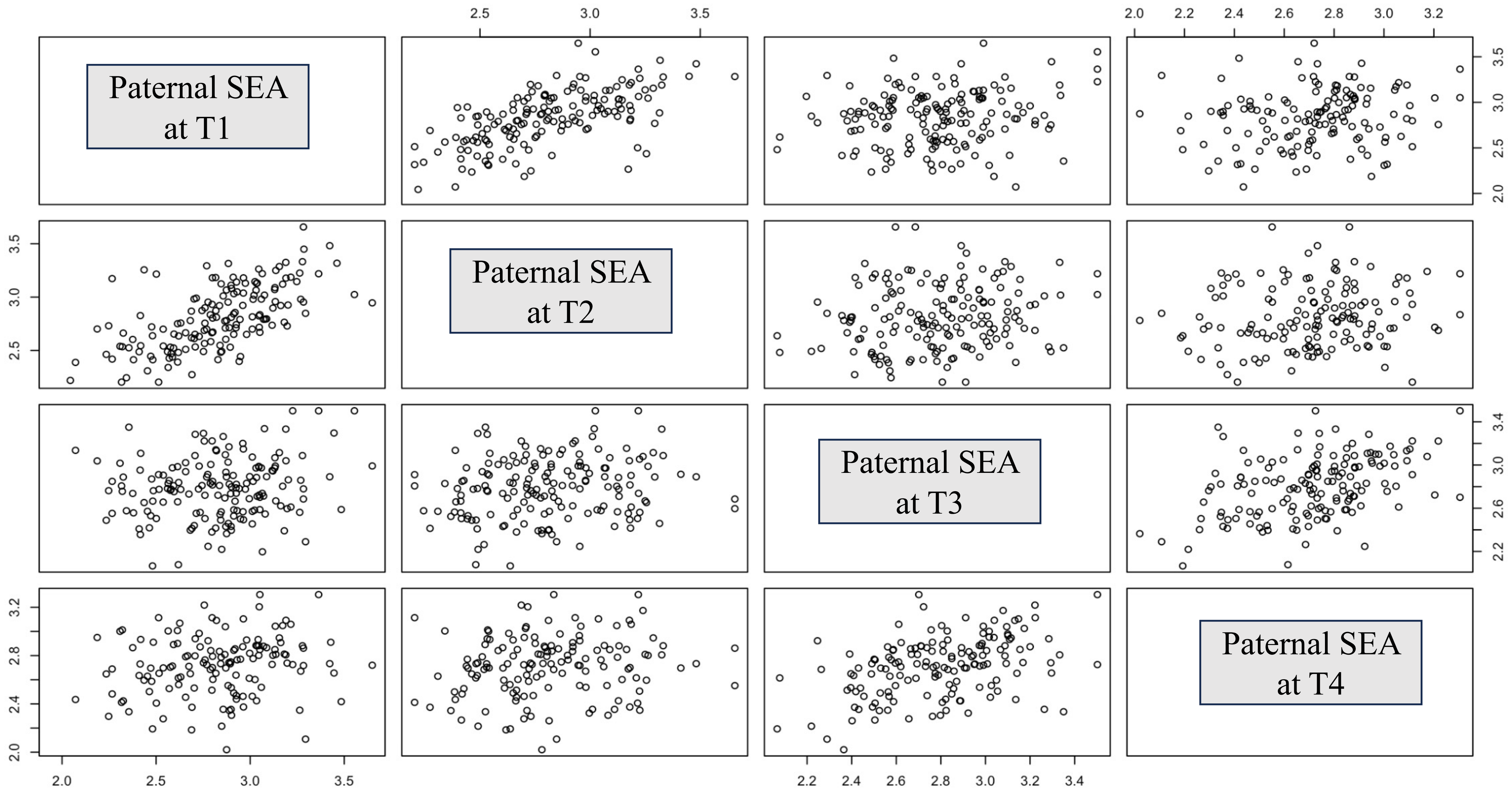

Relative Stability Fathers

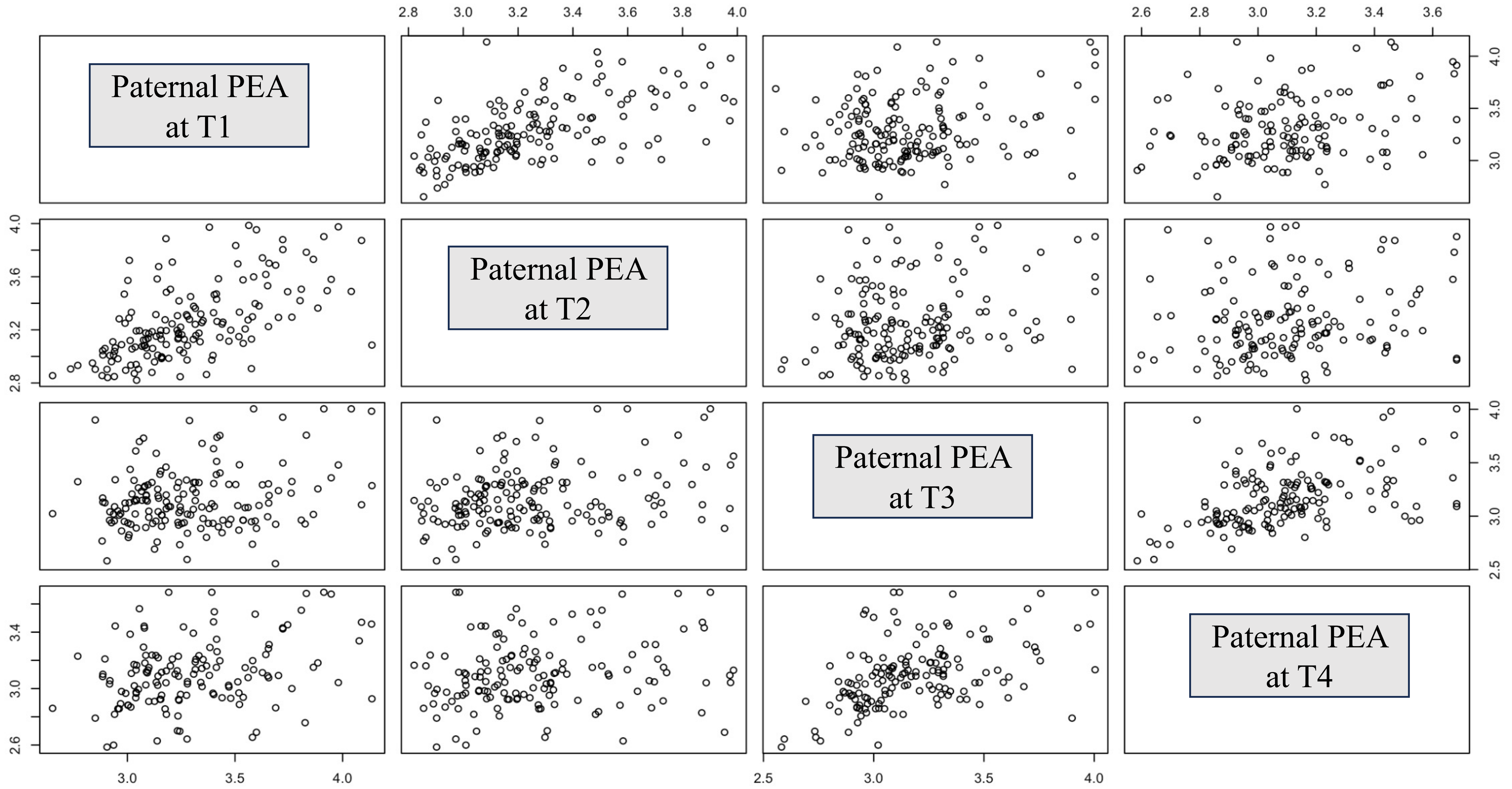

Relative Stability Fathers

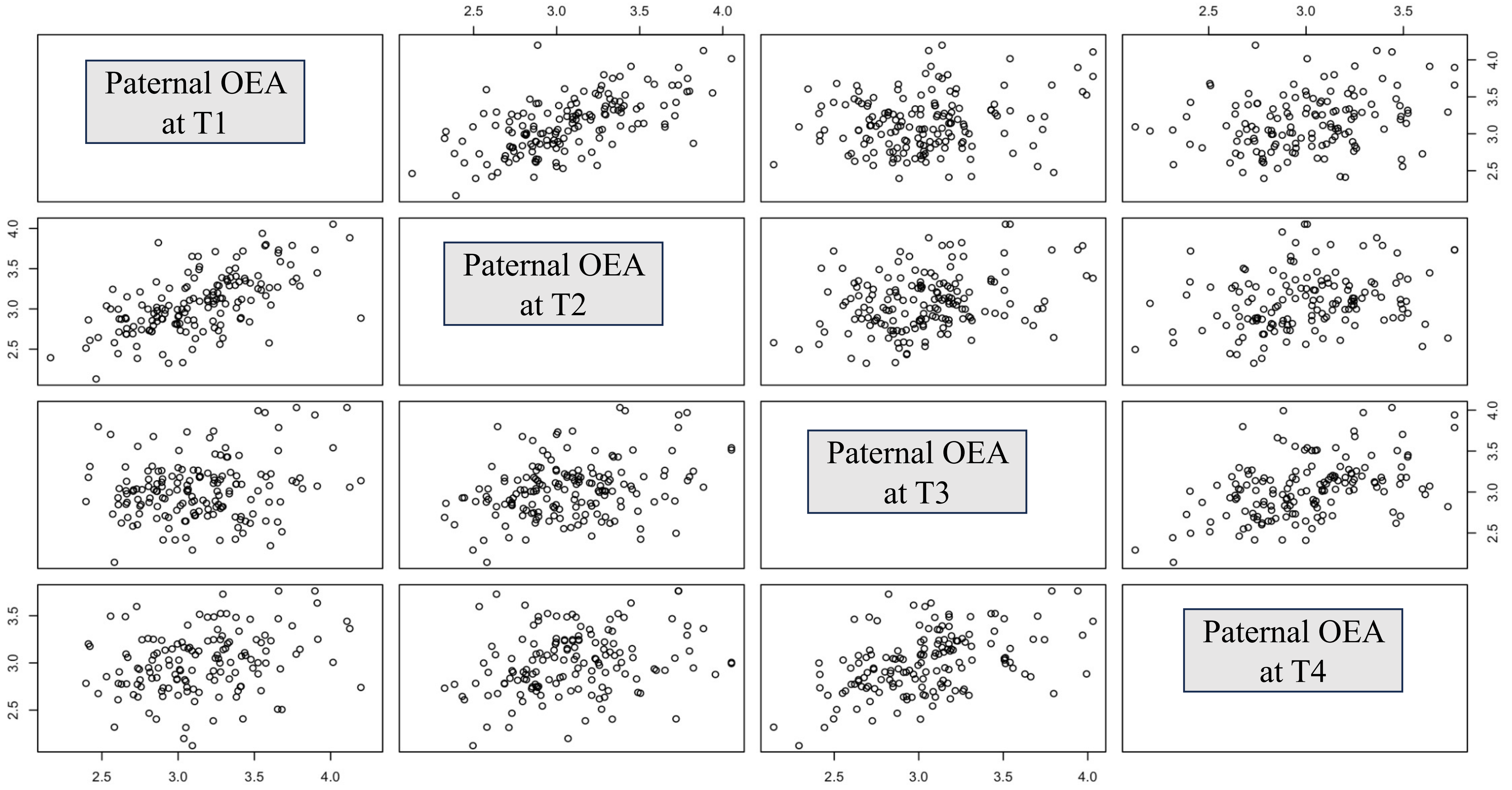

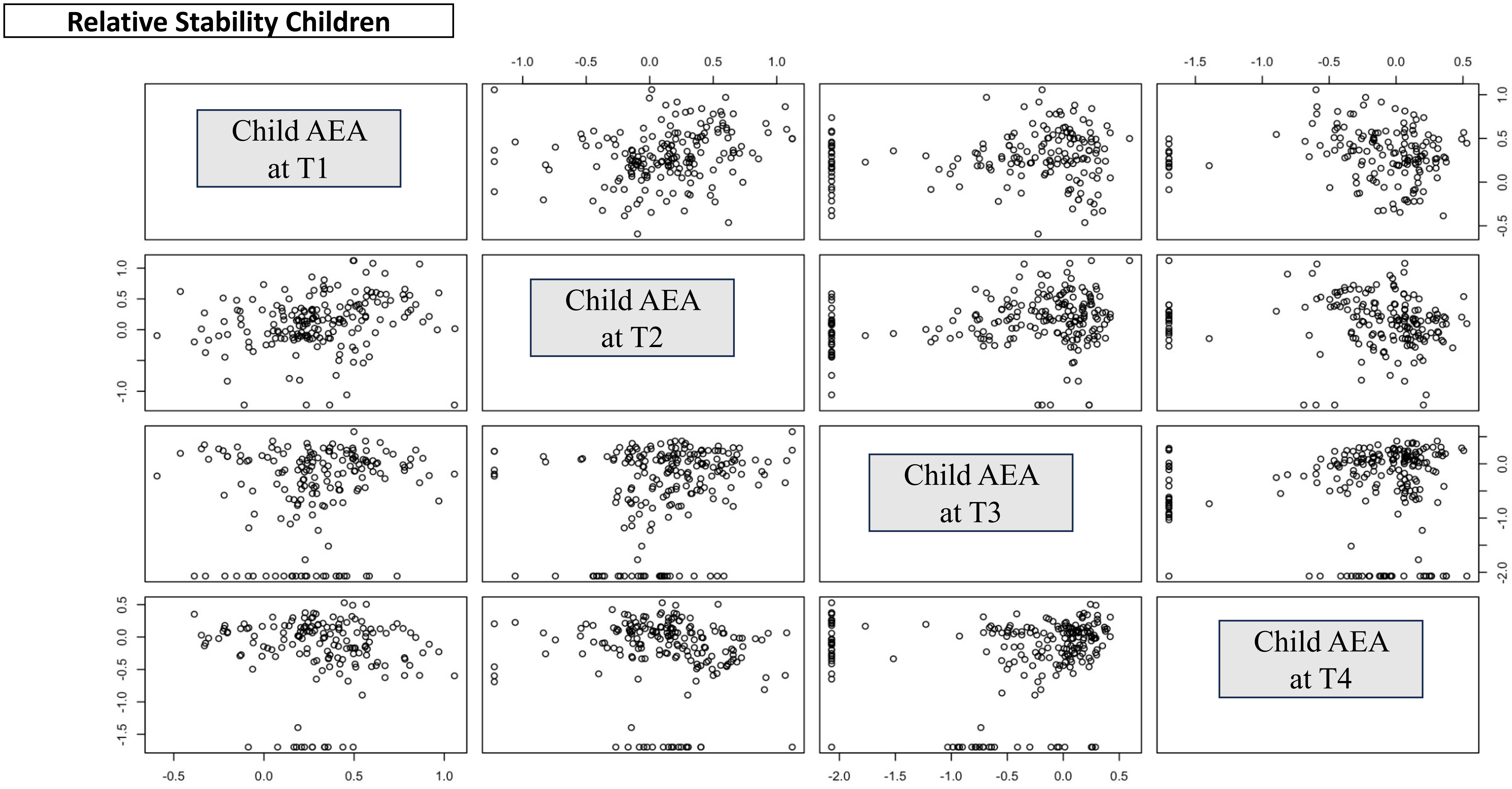
Relative Stability Children
Child AEA at T1
Child AEA at T2
Child AEA at T3
Child AEA at T4

Relative Stability Children

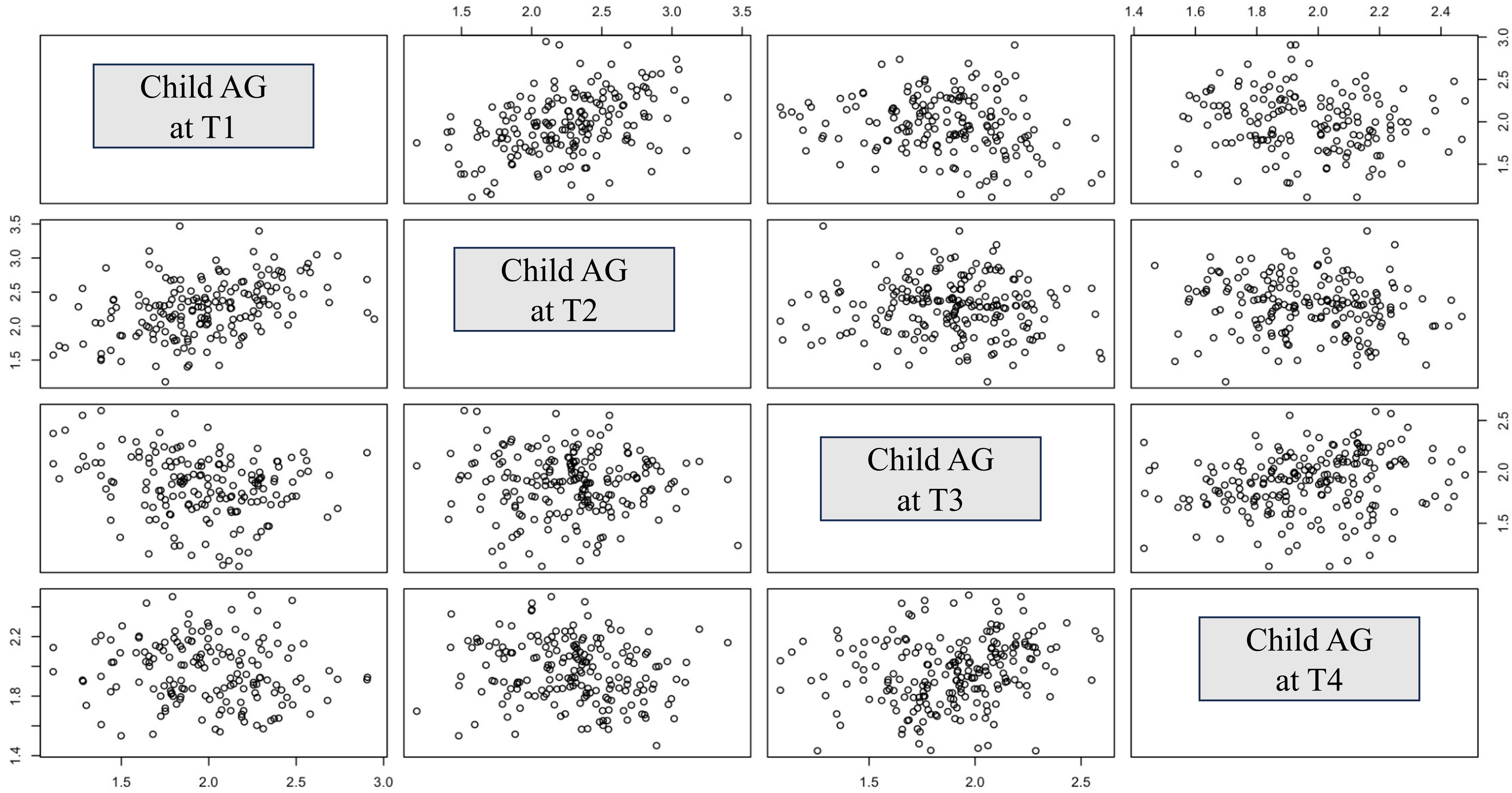

Relative Stability Children

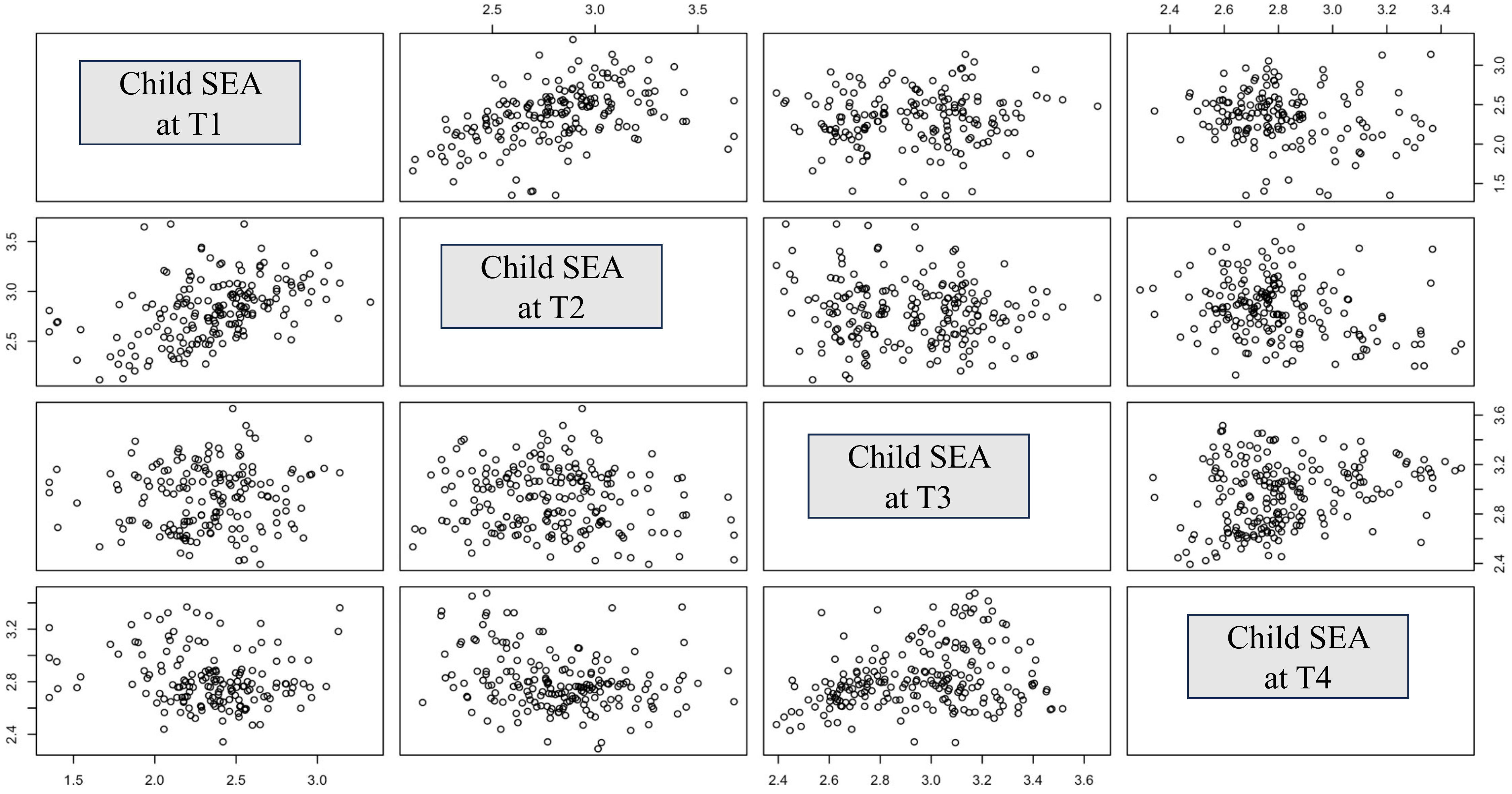

Relative Stability Children

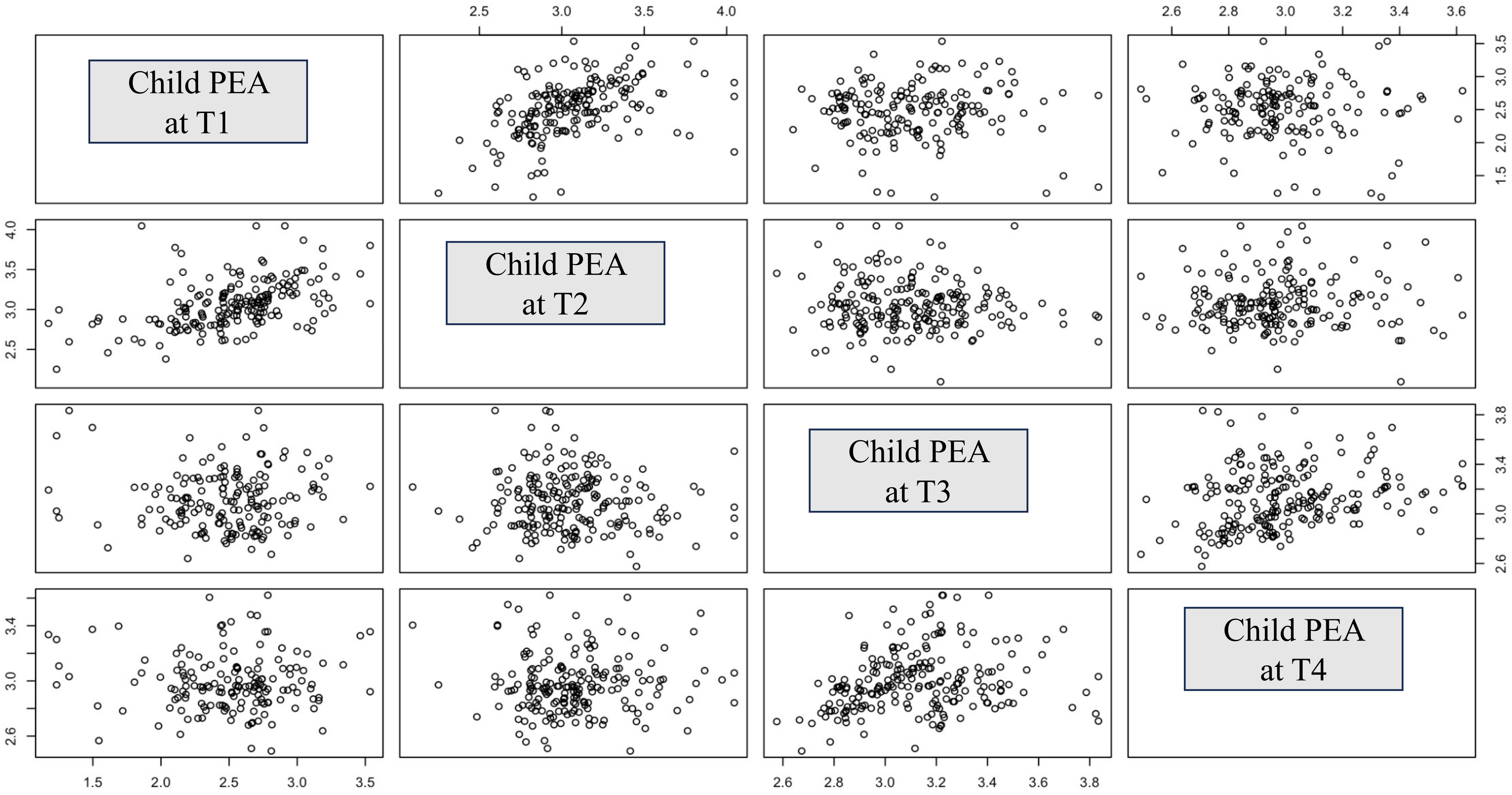

Relative Stability Children

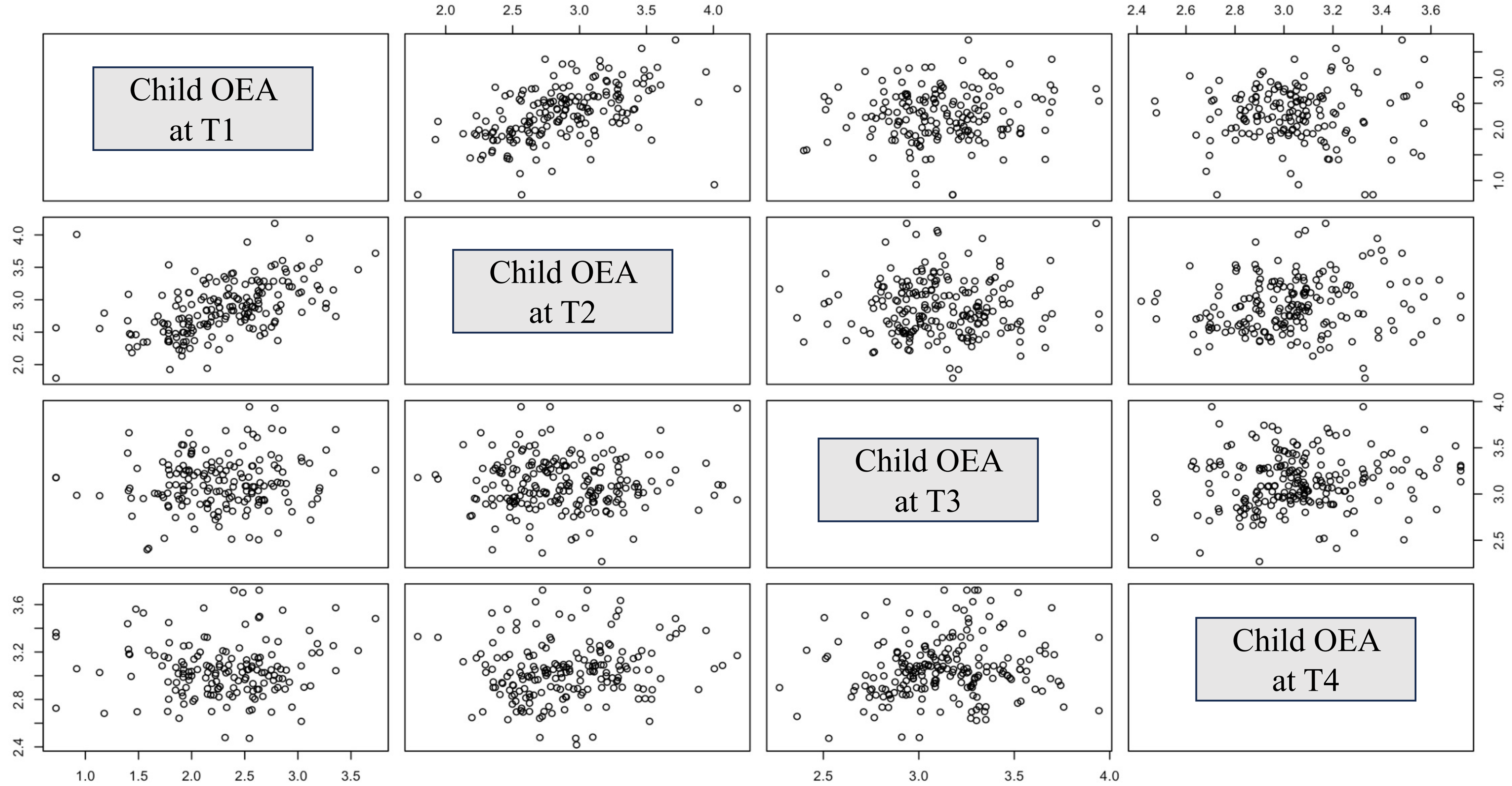

Inter-family association for hair AEA Mother-Child

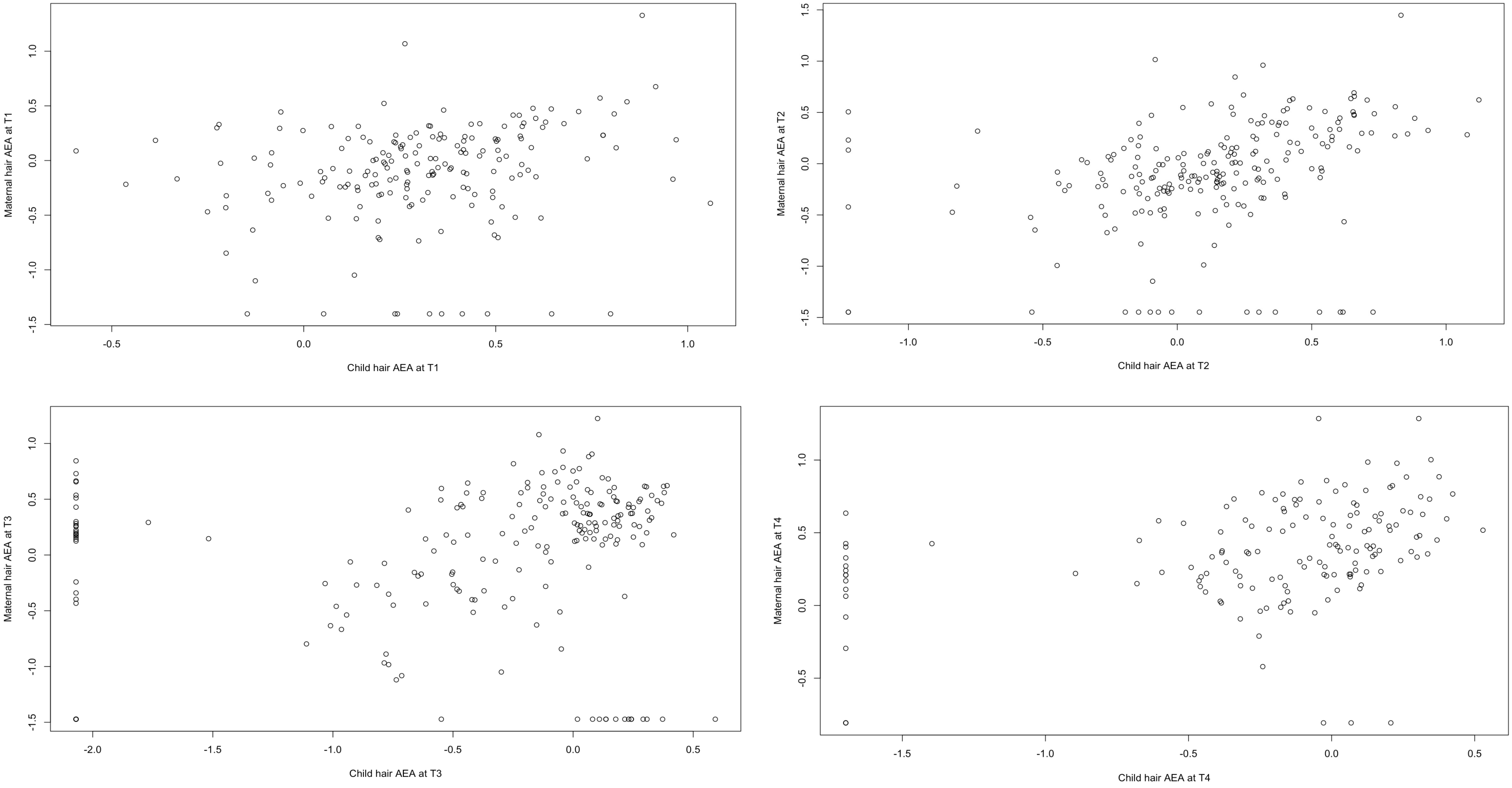

Inter-family association for hair 1-AG/2-AG Mother-Child

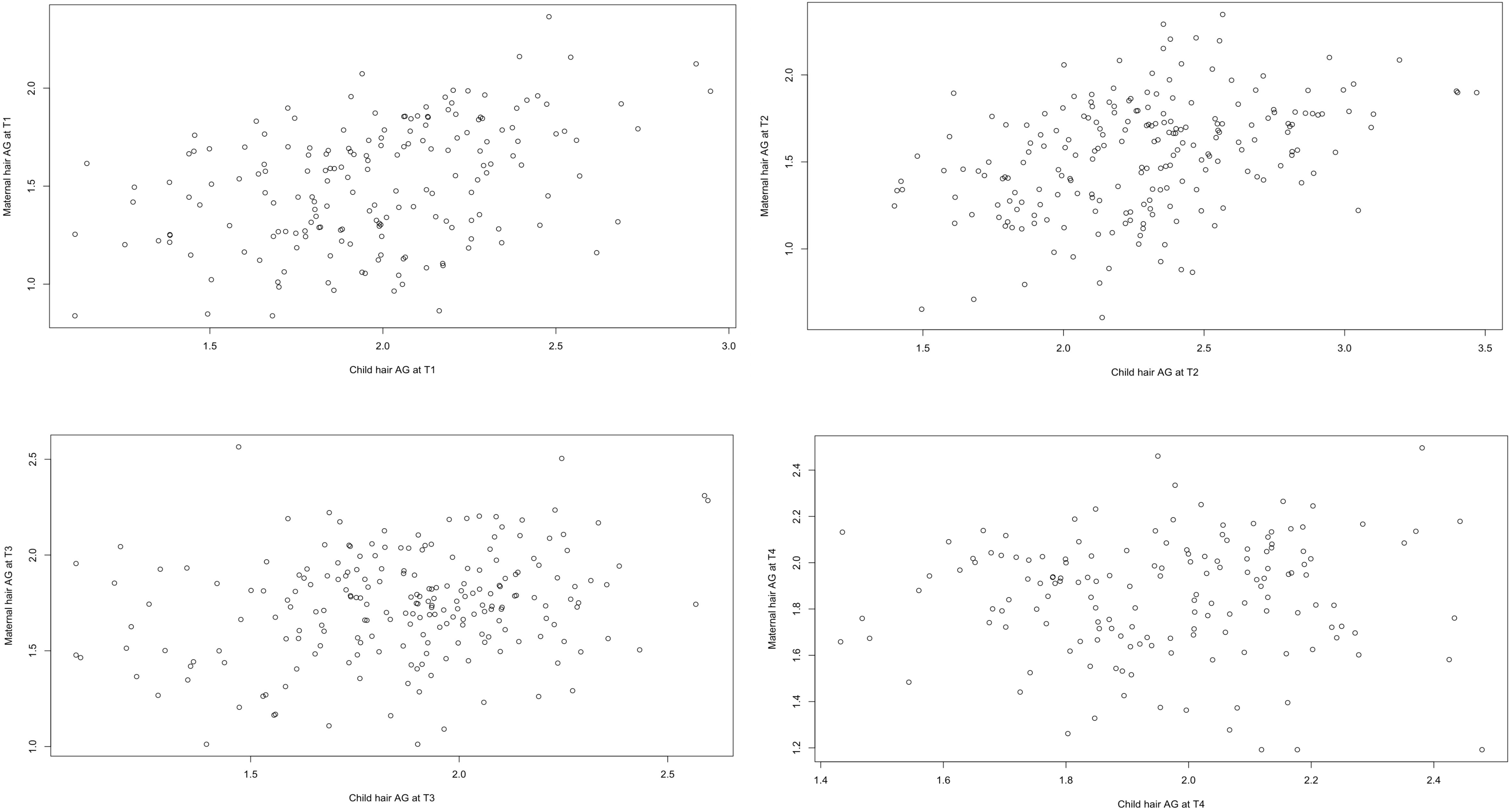

Inter-family association for hair SEA Mother-Child

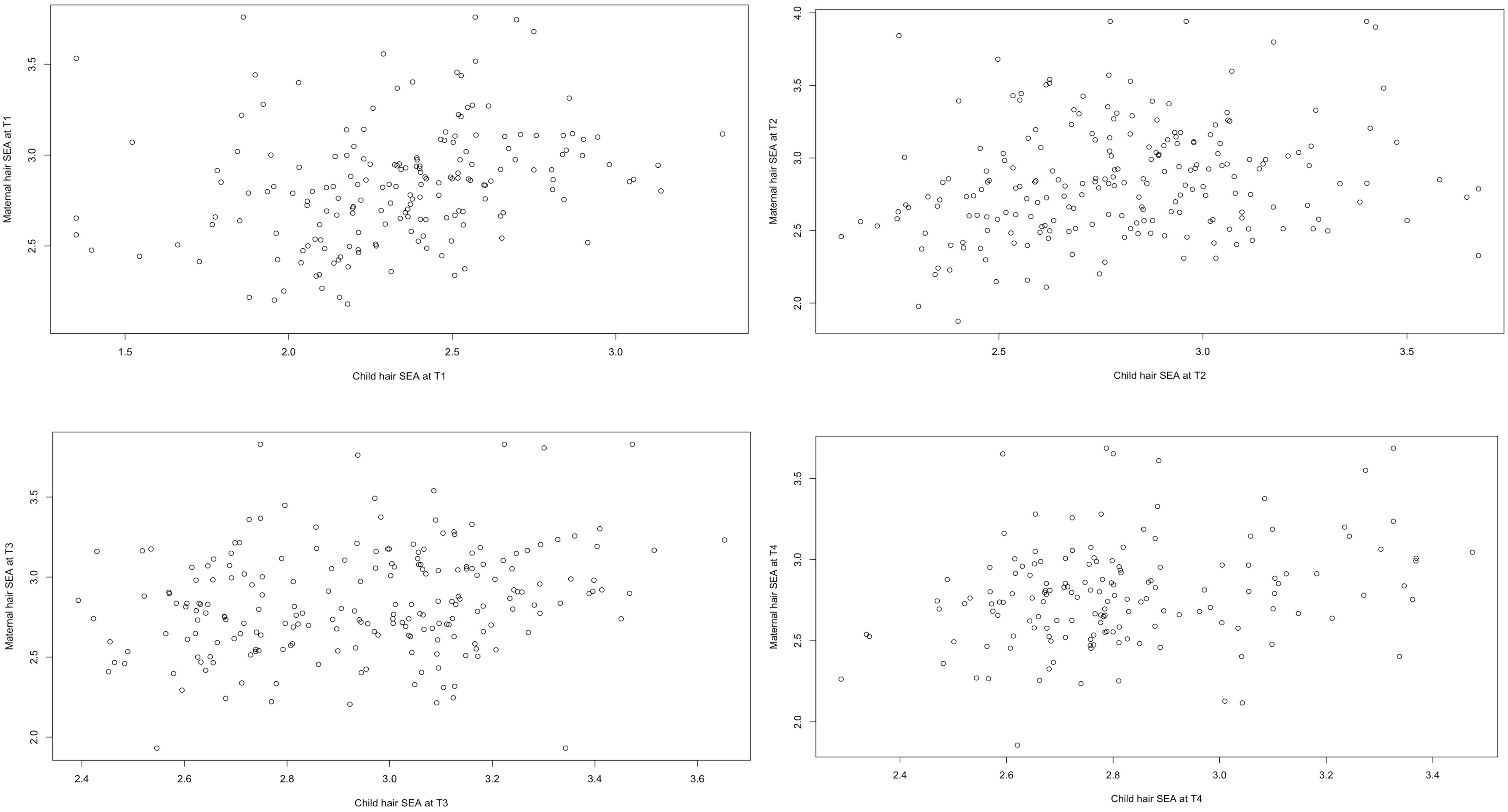

Inter-family association for hair PEA Mother-Child

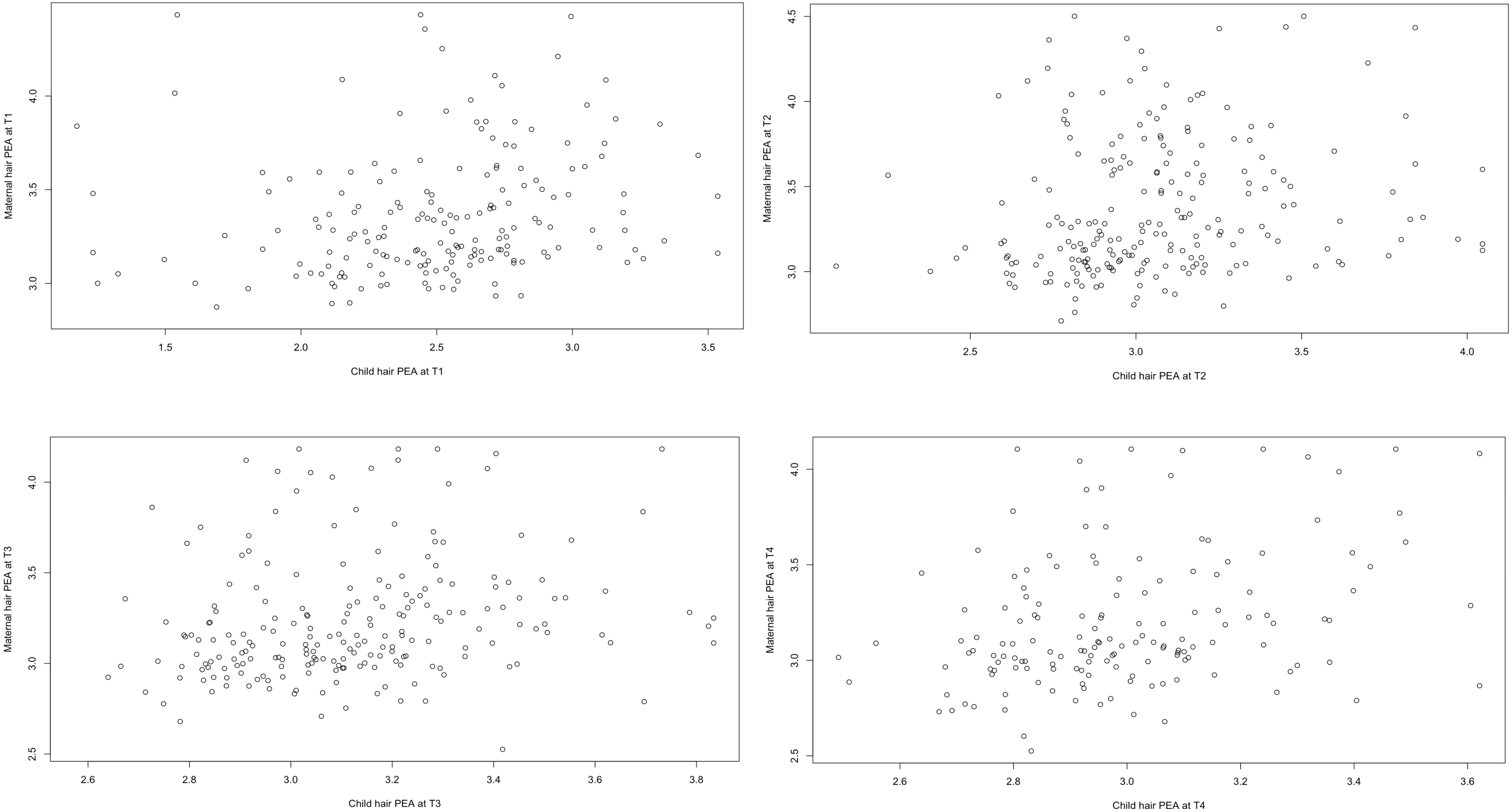

Inter-family association for hair OEA Mother-Child

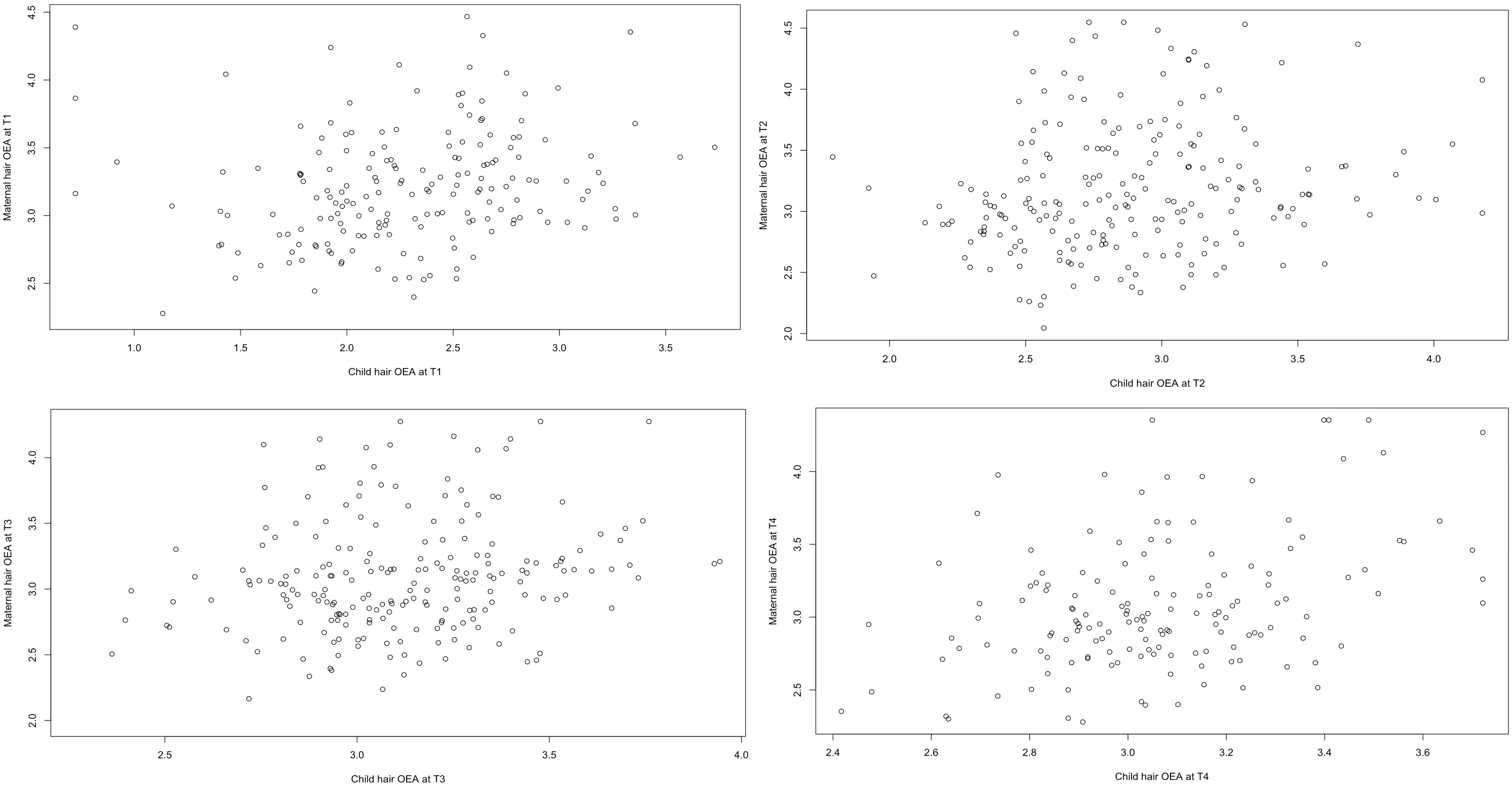

Inter-family association for hair AEA Father-Child

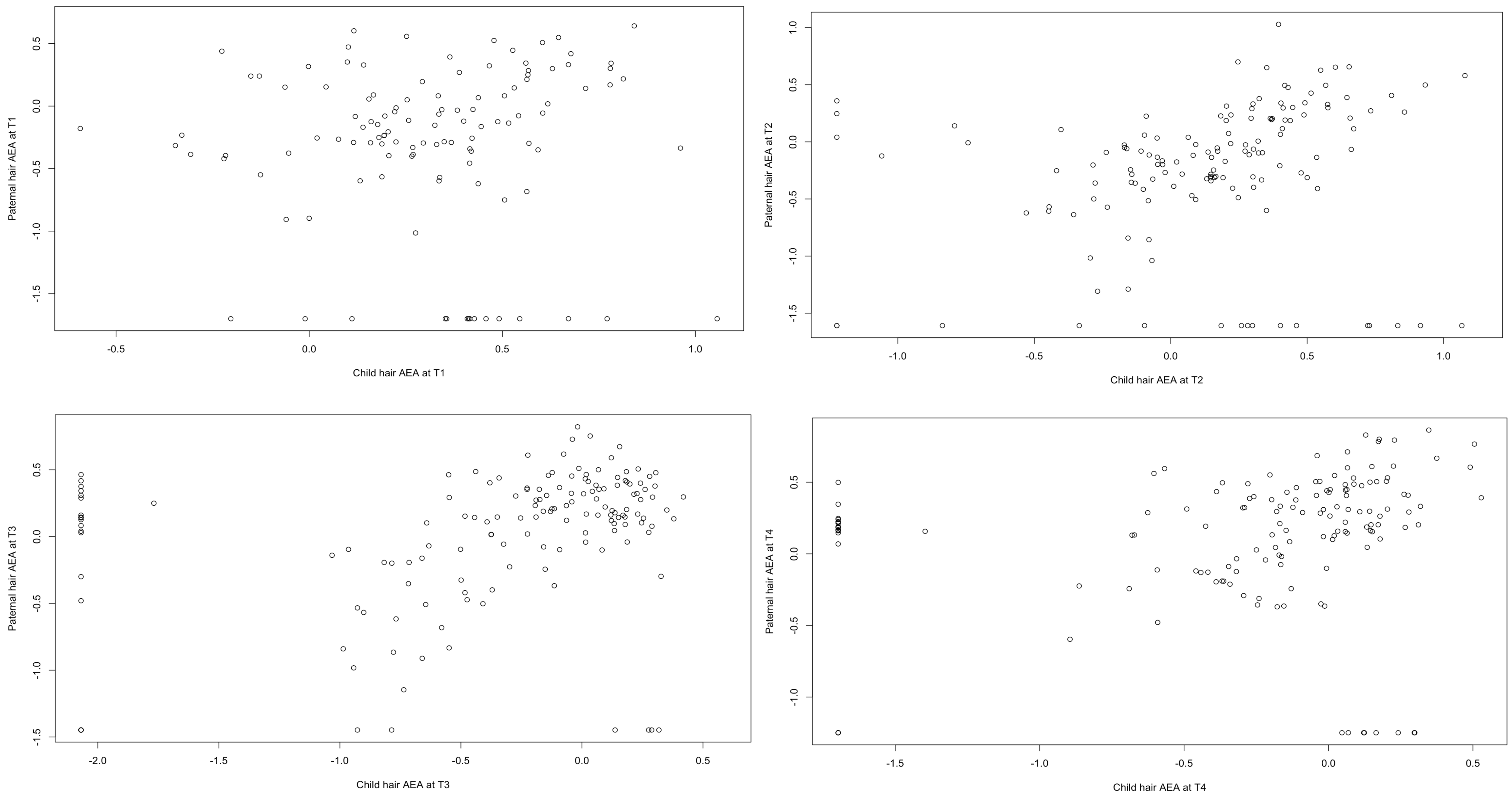

Inter-family association for hair 1-AG/2-AG Father-Child

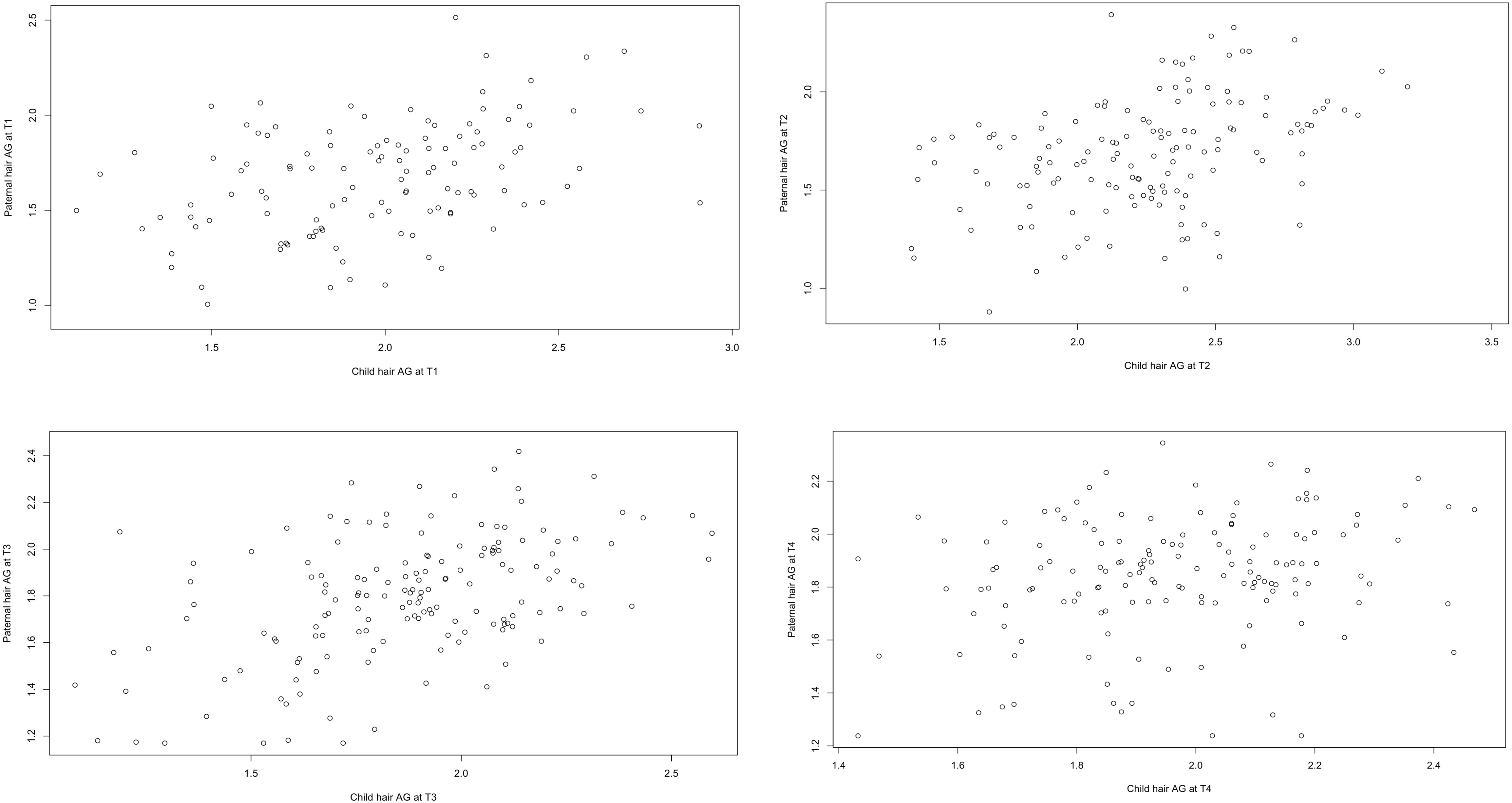

Inter-family association for hair SEA Father-Child

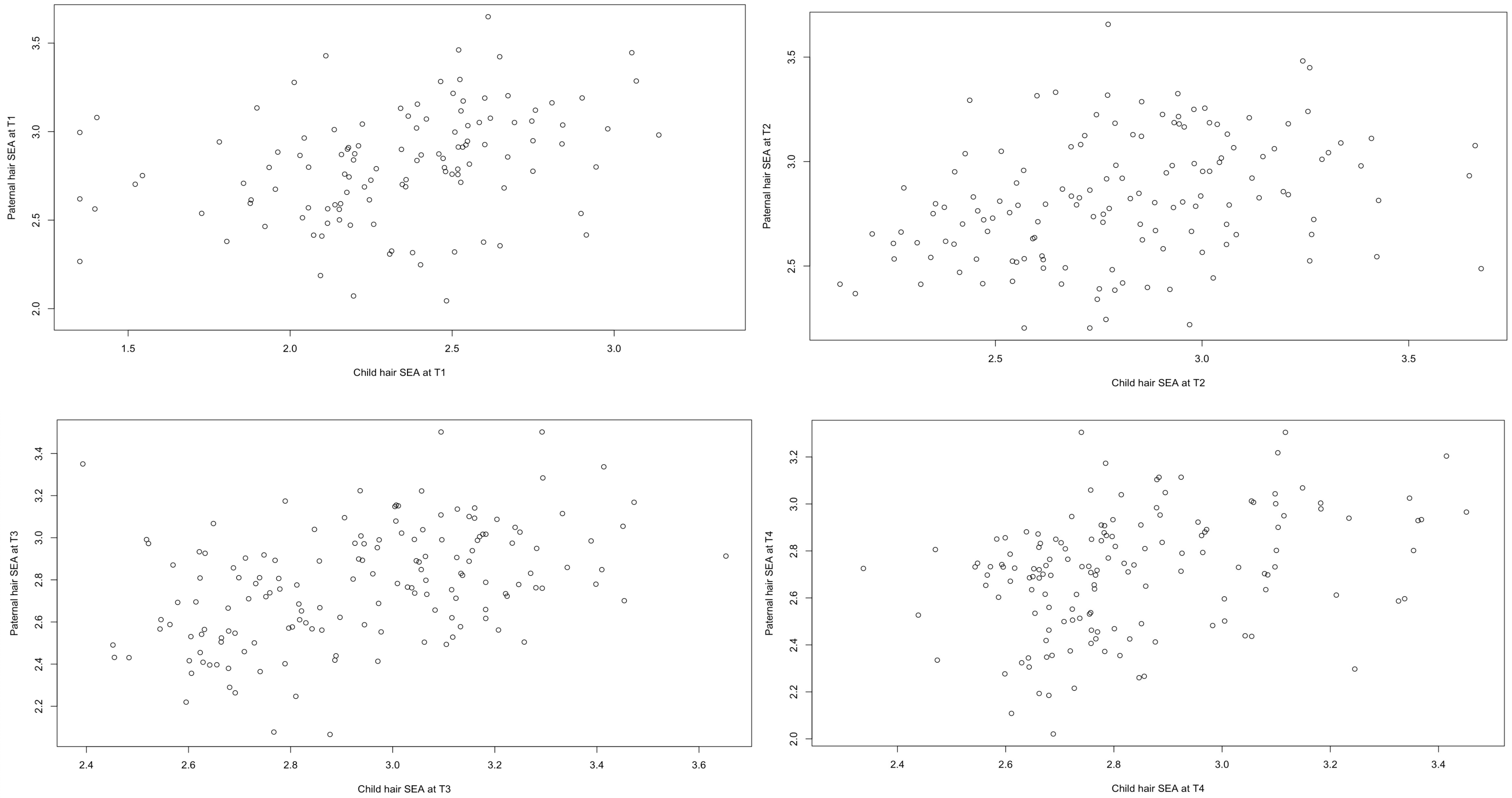

Inter-family association for hair PEA Father-Child

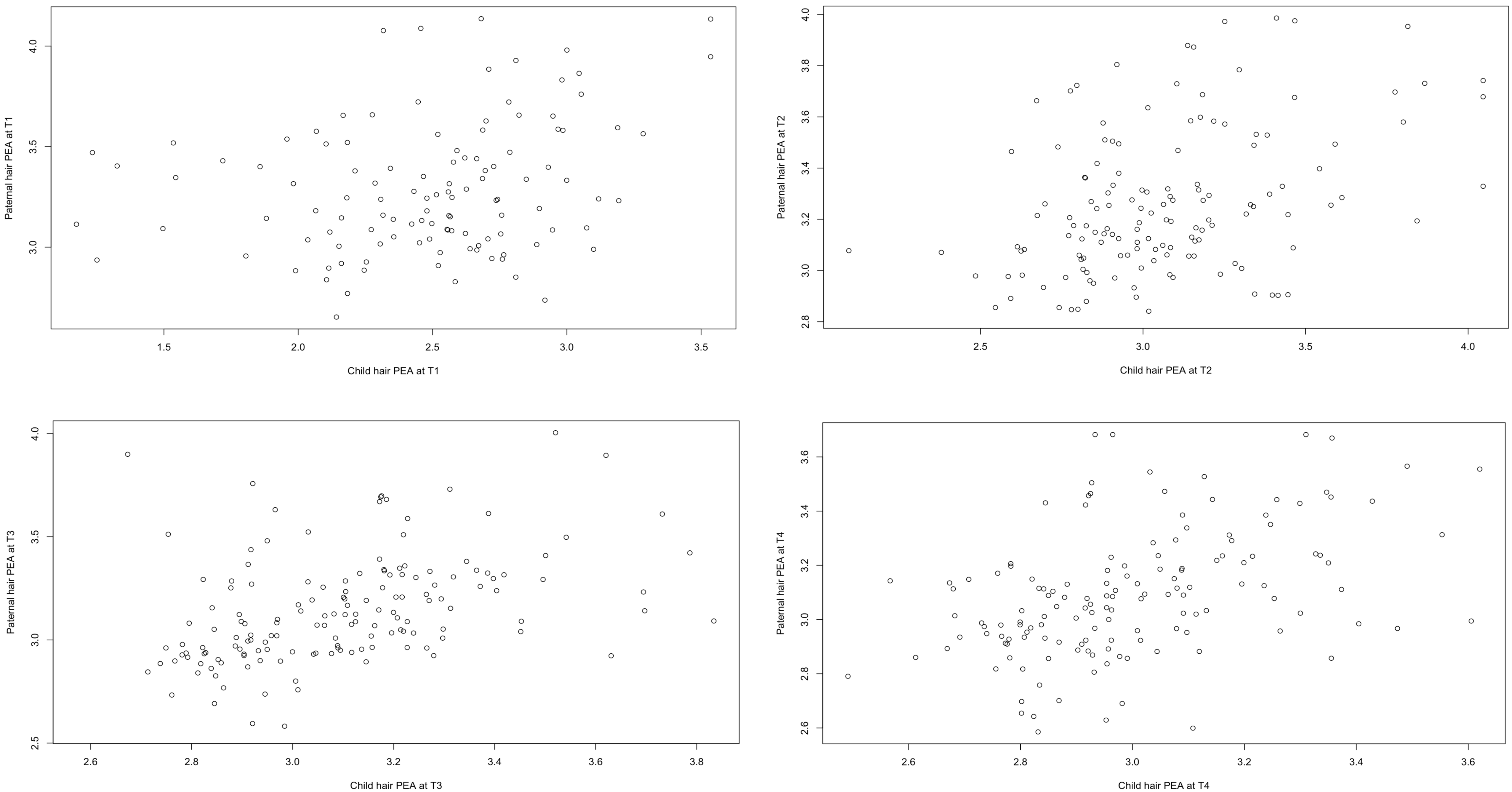

Inter-family association for hair OEA Father-Child

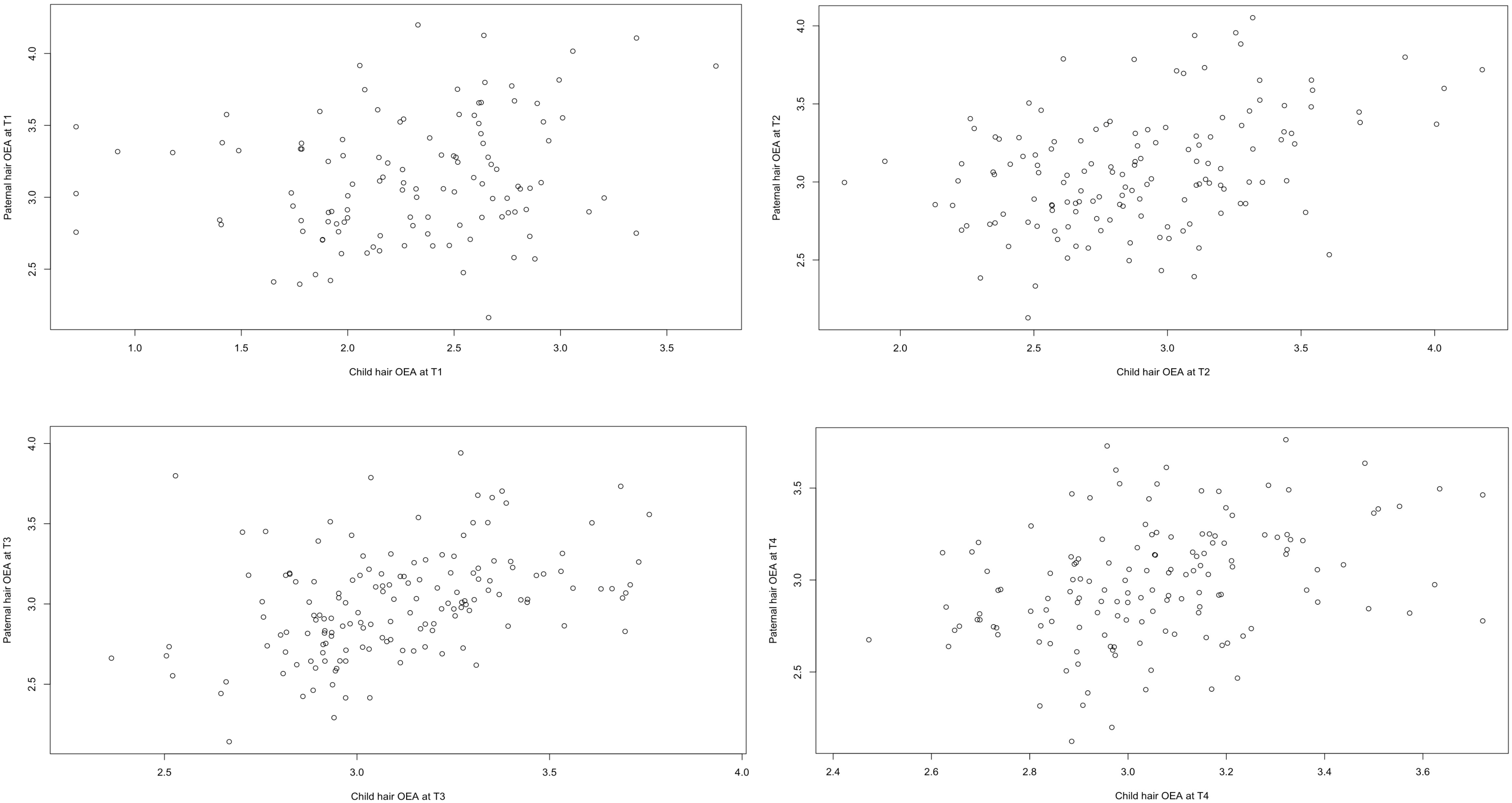

Inter-family association for hair AEA Father-Mother

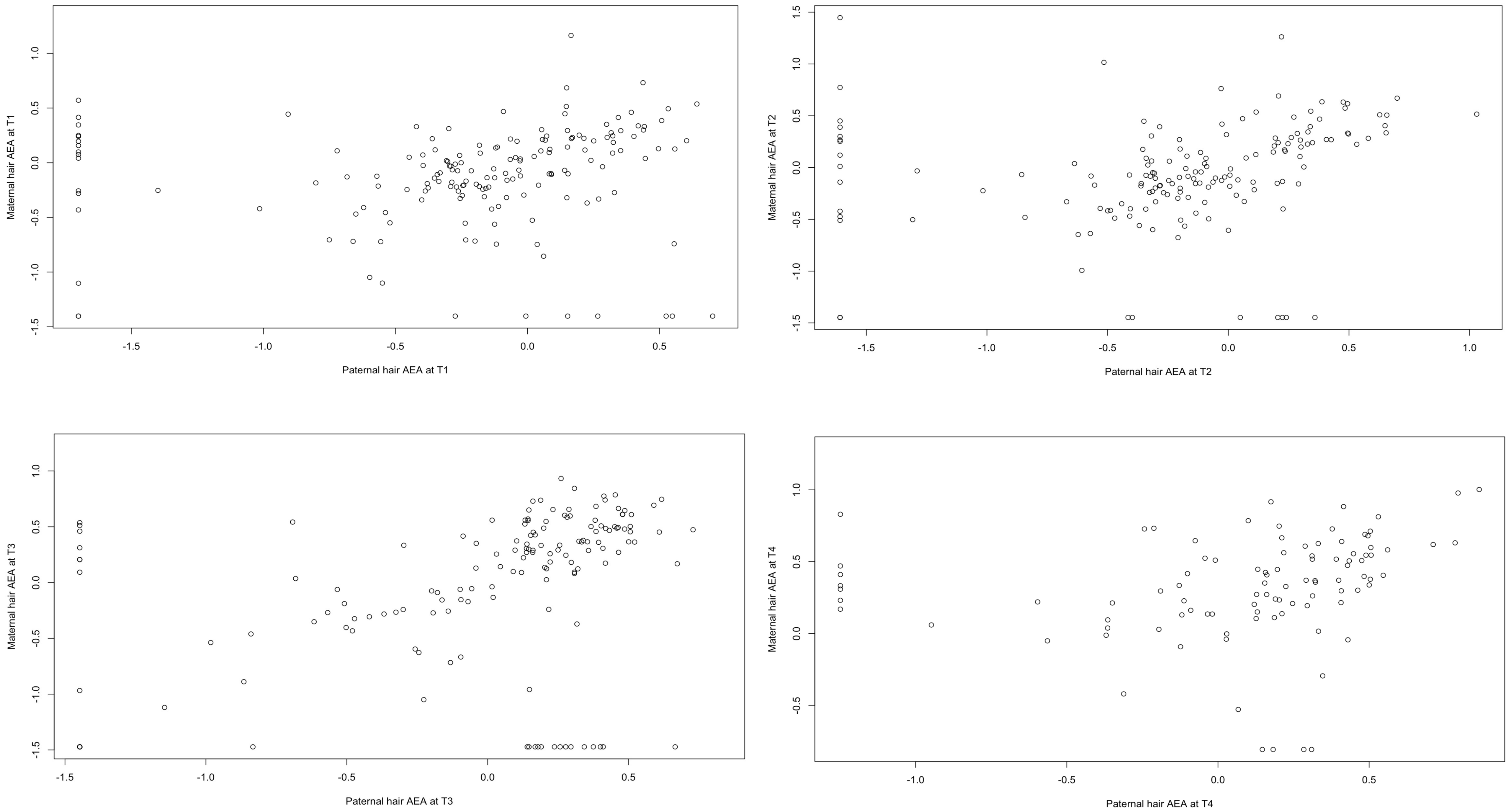

Inter-family association for hair 1-AG/2-AG Father-Mother

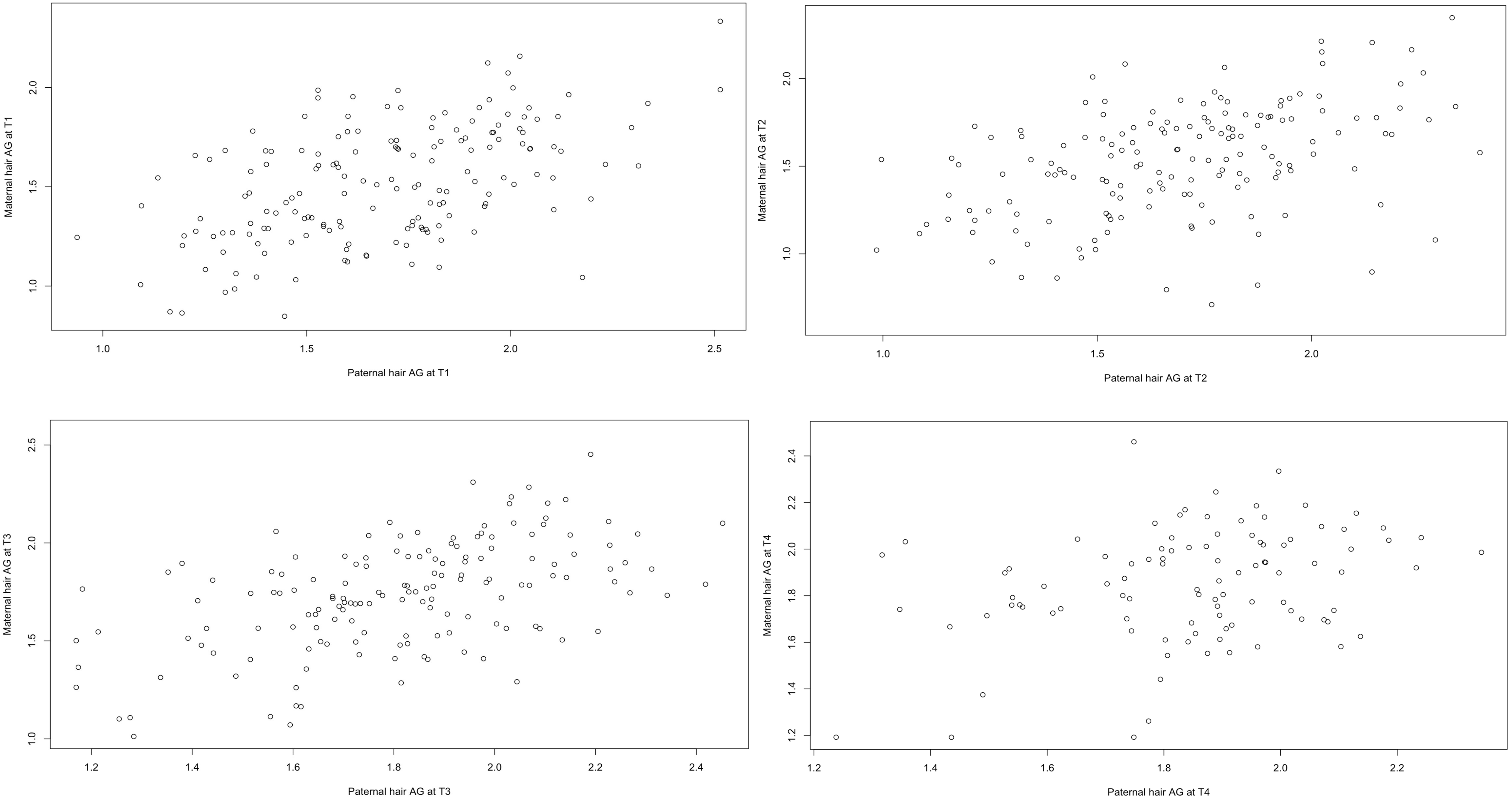

Inter-family association for hair SEA Father-Mother

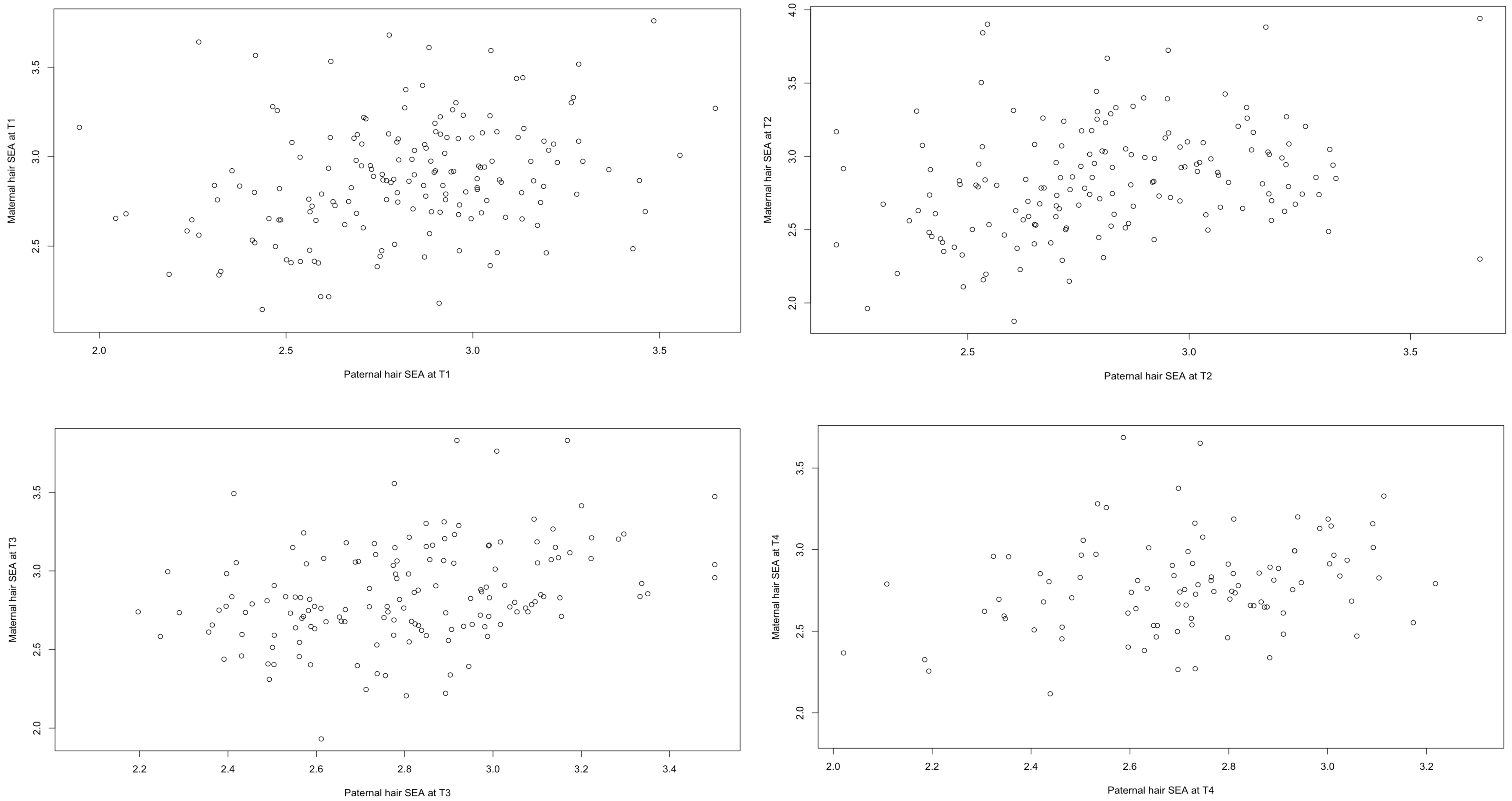

Inter-family association for hair PEA Father-Mother

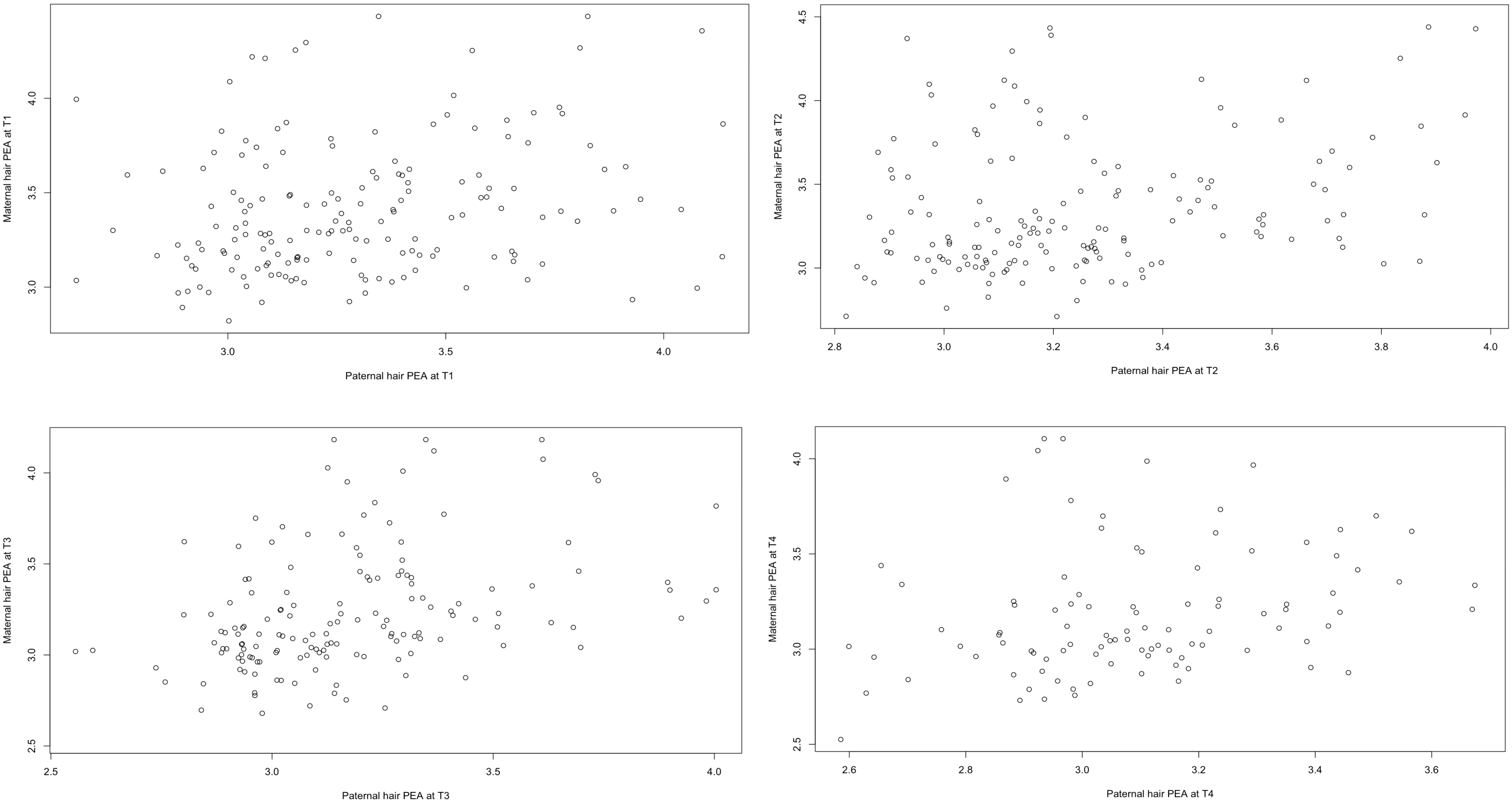

Inter-family association for hair OEA Father-Mother

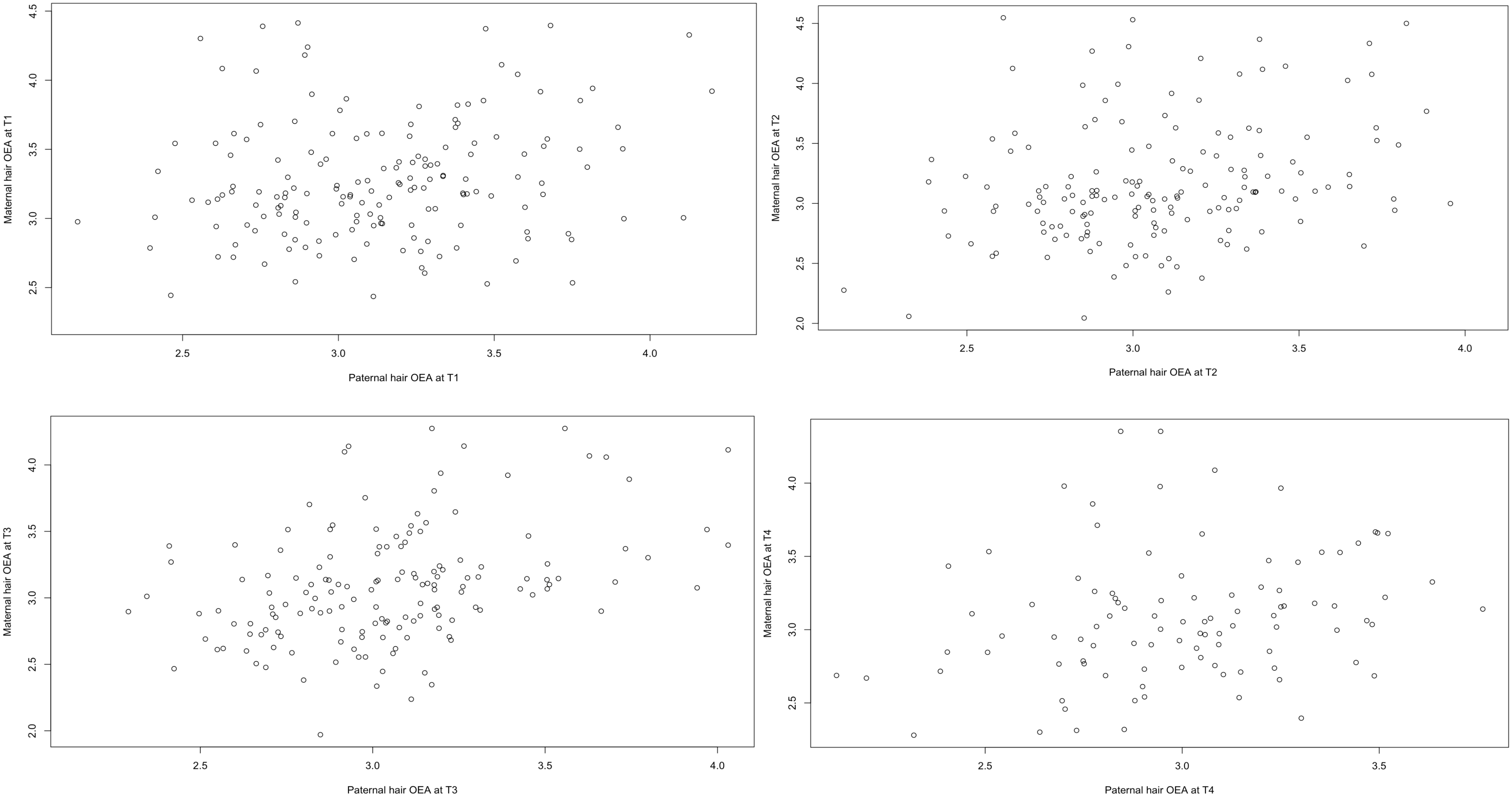

Supplement: Supplementary file 1 — Supplementary Information. [file 41598_2024_59818_MOESM1_ESM.pdf]
